# Protein separation and purification using inorganic oxide materials: from surface chemistry to biochemical applications

**DOI:** 10.1080/14686996.2026.2701640

**Published:** 2026-07-16

**Authors:** Shogo Kanoh, Kentaro Shiraki, Katsuya Kato, Atsushi Hirano

**Affiliations:** a Research Institute of Core Technology for Materials Innovation, National Institute of Advanced Industrial Science and Technology (AIST), Tsukuba, Ibaraki, Japan; b Institute of Pure and Applied Sciences, University of Tsukuba, Tsukuba, Ibaraki, Japan; c Chubu Center, National Institute of Advanced Industrial Science and Technology (AIST), Nagoya, Aichi, Japan

**Keywords:** Inorganic oxides, proteins, peptides, purification, adsorption, zirconia, titania, silica, magnetite, metal oxides

## Abstract

Inorganic oxide materials are widely used for protein and peptide separation because their surfaces support multiple types of interactions, including metal-centered coordination, electrostatic interactions, and both hydrophilic and hydrophobic interactions. This review summarizes the binding mechanisms between proteins/peptides and representative inorganic oxides—ZrO_2_, TiO_2_, SiO_2_, Fe_3_O_4_, Al_2_O_3_, ZnO, CeO_2_, SnO_2_, Nb_2_O_5_, Ta_2_O_5_, and Ga_2_O_3_. Particular emphasis is placed on the comparative analysis of adsorption mechanisms across these inorganic oxides and on factors such as crystal structure, surface acidity, and surface functionalization that influence adsorption selectivity. The review further discusses the effects of surface chemistry on purification performance and their combined effects on multi-step purification workflows. Recent advances in surface engineering and hybrid material design are highlighted to provide perspectives for the development of selective and mild protein purification processes. This framework offers guidance for the rational selection of inorganic oxide materials, enabling reproducible, selective, and mild purification of diverse proteins and peptides in biopharmaceutical applications.

## IMPACT STATEMENT

This review summarizes frameworks for protein and peptide purification using inorganic oxide materials. Surface chemistry, adsorption mechanisms, and workflow design are integrated to establish these frameworks including single-step and multi-step purification processes. These frameworks provide selective and robust protein and peptide purification.

## GRAPHICAL ABSTRACT

**Figure:**
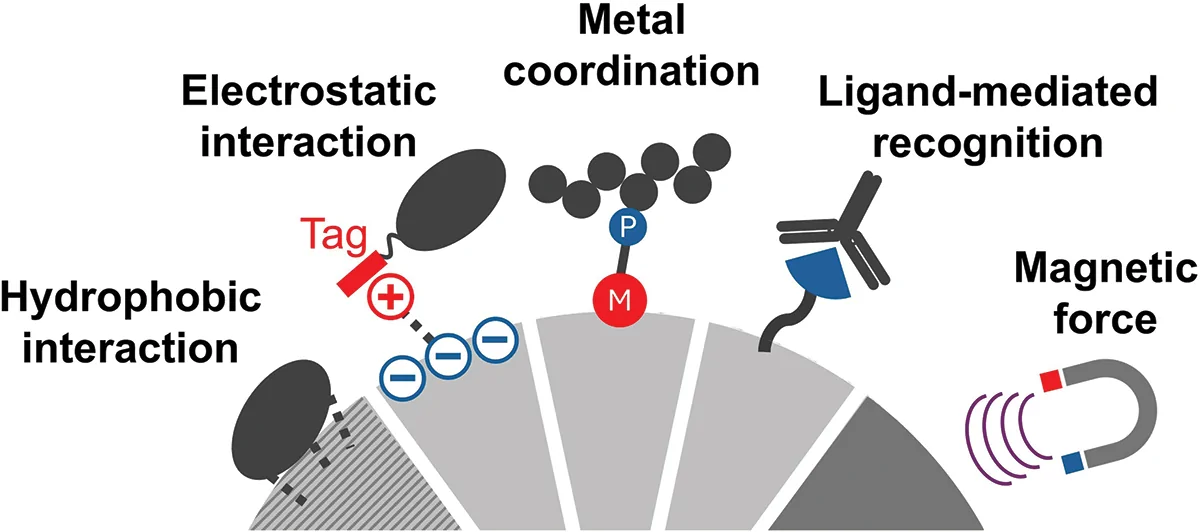

## Introduction

Proteins and peptides are central to medicine, biotechnology, and fundamental research [1–3]. They serve as therapeutic agents—such as monoclonal antibodies and peptide drugs—as well as industrial enzymes, diagnostic reagents, and model molecules used in molecular and cellular biological research [3,4]. However, their production processes typically generate complex mixtures containing host cells and cellular debris, host cell proteins (HCPs) and DNA, protein aggregates or cleavage products, and residual medium components supporting cell growth [4]. Efficient separation and purification are thus essential to ensure the safety, reproducibility, and cost-effectiveness of biopharmaceuticals [2,5]. High-yield and robust purification strategies are indispensable for both industrial bioprocessing and laboratory workflows that require well-defined protein preparations [1,5]. This demand drives the development of advanced materials and platforms for separation and purification.

Organic polymer-based media, such as agarose, are widely used in conventional protein purification [1,6]. Common formats include ion-exchange, reversed-phase, size-exclusion, and affinity chromatography, which exploit electrostatic, hydrophobic, and specific ligand–protein interactions [1,7]. They are widely used at both laboratory and industrial scales, but organic polymer-based media have several limitations. For example, their chemical and mechanical stability may be insufficient under harsh conditions, reducing reusability and long-term performance [8–10]. Additionally, gel swelling in solution can affect column packing, flow rate, and pressure [11]. Thus, alternative platforms with greater chemical robustness and operational durability, are needed for reliable and reproducible protein purification.

Inorganic oxides are promising candidates for versatile and robust alternatives. Representative materials such as ZrO_2_, TiO_2_, SiO_2_, and Fe_3_O_4_ exhibit high chemical and mechanical stability and can withstand oxidative and other harsh conditions that would damage conventional organic polymer-based medium [12–14]. Most of these inorganic oxides possess surface hydroxy groups and acid–base active sites, enabling multiple interaction modes with adsorbates [15]. ZrO_2_ and TiO_2_ offer selective interactions with proteins and peptides through high-density metal centers [16]. SiO_2_ provides complementary selectivity for proteins and peptides through hydroxy-mediated electrostatic interactions and hydrophobic interactions arising from surface siloxane regions [17]. Magnetic oxides, such as Fe_3_O_4_, enable rapid and straightforward recovery of adsorbed proteins and peptides, facilitating high-throughput purification workflows [18]. Collectively, these characteristics of inorganic oxides offer versatile and robust platforms for protein and peptide purification across diverse biological and industrial applications.

This review provides a comprehensive overview of inorganic oxide materials for protein and peptide separation and purification, with particular emphasis on structure–function relationships, material-specific adsorption mechanisms, and selective enrichment strategies. While previous studies have primarily focused on individual materials or separation applications, comprehensive comparisons of adsorption mechanisms, surface chemistry, and separation performance across different inorganic oxides remain limited. This review provides a comparative perspective on inorganic oxide materials for protein and peptide separation and purification, including a comparison of key factors governing adsorption behavior, such as crystal structure, surface acidity, and surface functionalization. Particular emphasis is placed on elucidating how these factors govern protein and peptide adsorption selectivity. The discussion includes the advantages, limitations, and application scopes of representative inorganic oxides for phosphorylated peptide enrichment, tagged protein purification, antibody purification, and other biomolecule purification processes. By integrating mechanistic insights with recent advances in surface engineering and hybrid material design, this review provides guidance for rational material selection and offers perspectives for purification workflow development in future protein and peptide purification applications.

## General principles of protein and peptide adsorption on inorganic oxides

Inorganic oxides provide chemically diverse interfaces that enable protein and peptide adsorption through multiple interaction modes. Their surface metal centers act as Lewis-acidic sites with varying affinities for oxygen-donor ligands, while surface hydroxy groups impart amphoteric character through protonation and deprotonation equilibria. These properties generate surface charge distributions that depend on solution pH and ionic strength, thereby modulating adsorption strength and reversibility. Surface modification and particle morphology further regulate the accessibility and density of adsorption sites, defining the overall adsorption landscape in oxide-based separation systems. For comparison across materials, Table 1 summarizes the representative sample formats, surface properties, functional groups, and target biomolecules of major inorganic oxides.

**Table 1.** Representative formats, surface properties, functional groups, and target biomolecules of inorganic oxides.

	Sample formats	Surface properties	Functional groups	Target biomolecules
ZrO_2_	Monoclinic phase [19–21]/ Nanoparticles [22]; Mesoporous beads [23,24]; Microtips [25]	Lewis-acidic Zr^4+^ surface sites [26] Interfacial hydration layers [27]	Aliphatic hydroxy Acid (e.g. β-hydroxypropanoic acid) [28] Phosphonate compounds (e.g. ethylenediamine tetra(methylene phosphonic acid) (EDTMP)) [19,21,29] Phosphate compounds (e.g. Phosphate) [20]	Phosphorylated peptides [22–25,28] His-tagged proteins [21] Antibodies (IgG, IgM, IgA) [19,20,30–34]
TiO_2_	Anatase phase [29,35–37]/ Nanoparticles [35]; Mesporous beads [36,38]; Microtips [25]	Lewis-acidic Ti^4+^ surface sites [39] Interfacial hydration layers [40,41]	Amine [35] Aliphatic hydroxy Acid (e.g. Lactic acid) [28] Phosphonate (e.g. alendronate sodium trihydrate, nitrilotri(methylphosphonic acid) (ATMP)) [29]	Phosphorylated peptides [16,28,29,35,36,38,42–45] Highly Similar Proteins (separation of proteins which similar sizes and net charges) [37]
SiO_2_	Amorphous silica [17,46–49]; α-quartz [50–53]/Porous silica gel [17]; Porous particles [46,54–57]; Nanoparticles [48,58]; Core–shell magnetic particles [59]	Comparatively low intrinsic Lewis acidity [60] High silanol density [61] Deprotonatable silanol groups [62] Hydrophilic hydroxylated surface [61] Hydration-dependent surface hydrophobicity [61]	IDA/NTA-metal complexes (Co^2+^, Cu^2+^) [59,63] ZrO_2_/TiO_2_ coatings [48,58] Ion-exchange ligands (e.g. morpholine, tetrazole) [64,65] Hydrophobic ligands (e.g. C18, cholesterol) [55,66] Hydrophilic ligands (e.g. glutathione) [49,56] Dye ligands (e.g. Cibacron Blue F3G-A) [54] Boronic acid compounds [67] Polymer coatings (e.g. cationic/anionic polymers) [68,69] Affinity biomolecules (e.g. protein A, concanavalin A, affinity peptide) [57,70–72]	Recombinant/model proteins (e.g. lysozyme, cytochrome C) [17,46,54,55,65,68,69,73] Tagged proteins (e.g. Si-tag, His-tag) [47,50–53,59,63,74,75] Phosphorylated peptides [48,58] Glycopeptides/proteins [49,56,67,71] Antibodies (IgG) [57,70,72]
Fe_3_O_4_	Inverse spinel structure [76]/ Nanoparticles [18,76–80]; Core–shell microspheres [81–85]	Lewis-acidic surface Fe cation sites [86] Amphoteric surface hydroxyl groups [87] Magnetic separability [18,77–79,81,84,85,88]	Metal ion-coordinating polymer layers [78,82,88] Boronic acid ligands [79,81] SiO_2_/TiO_2_/ZrO_2_ coatings [81,83–85] Affinity biomolecules (e.g. protein A, enzyme) [80,89]	Phosphorylated peptides [83,84] His-rich proteins [82] Tagged proteins (e.g. CotB1p-tag, His-tag) [18,77,78,85,88] Glycopeptides/proteins [79,81] Antibodies (IgG) [70,80]
Al_2_O_3_	γ-Al_2_O_3_ [90]/ Packed chromatographic particles [90]; Magnetic core–shell microspheres [91]	Lewis-acidic Al^3+^ surface sites [92] Amphoteric surface hydroxyl groups [93]	Octadecyl and polybutadiene coatings [94,95]	Phosphorylated peptides [91] Strongly basic proteins [90]
ZnO	Wurtzite ZnO [96] / Nanoparticles [96–100]	Amphoteric surface hydroxyl groups [101] Hydrogen bonding and van der Waals contributions [96,100]	–	–
CeO_2_	Face-centered cubic phase [102] / Nanoparticles [97,98,103]; Monodisperse-porous microspheres [102]	Lewis-acidic Ce^4+^ surface sites [104,105] Amphoteric surface hydroxyl groups [106]	–	Phosphorylated peptides/proteins [102,103]
SnO_2_	Not specified / Porous microspheres [107,108]	Lewis-acidic Sn^4+^ surface sites [109]	–	Phosphorylated peptides [107,108]
Nb_2_O_5_	Not specified / Powder [110]	Lewis-acidic Nb^5+^ surface sites [111]	–	Phosphorylated peptides [110]
Ta_2_O_5_	Not specified /Core–shell magnetic microspheres [112]	Lewis-acidic Ta^5+^ surface sites [113]	–	Phosphorylated peptides [112]
Ga_2_O_3_	Not specified / Core–shell magnetic particles [114]	Lewis-acidic Ga^3+^ surface sites [115,116]	–	Phosphorylated peptides [114]

Lewis acidity is a key factor governing the adsorption behavior of solutes on inorganic oxide surfaces. Surface metal centers such as Zr^4+^ and Ti^4+^ act as Lewis-acidic electron-pair acceptors that coordinate oxygen-donor ligands, which function as Lewis bases. ZrO_2_ and TiO_2_ are representative inorganic oxides widely used for coordination-mediated adsorption and separation because of their strong Lewis-acidic surface sites [26,39,117]. Similar coordination behavior has also been reported for other inorganic oxides containing Lewis-acidic metal centers, including Fe_3_O_4_, Al_2_O_3_, CeO_2_, ZnO, Nb_2_O_5_, Ta_2_O_5_, SnO_2_, and Ga_2_O_3_ [92,104,105,109,111,113,115,116,118–120]. In contrast, SiO_2_ generally exhibits weak or negligible Lewis acidity compared with many transition-metal oxides [60]. Among Lewis-basic ligands, phosphate groups readily interact with coordinatively unsaturated metal centers on oxide surfaces through metal–oxygen coordination. These Lewis acid–base interactions underpin phosphate-related adsorption on inorganic oxide surfaces and contribute to the selective enrichment of phosphorylated biomolecules.

Surface charge on inorganic oxide surfaces is commonly governed by protonation and deprotonation equilibria of surface hydroxyl groups and is commonly characterized by the point of zero charge (PZC). SiO_2_ generally exhibits low PZC values because of the acidic nature of surface silanol groups, whereas Al_2_O_3_, ZnO, and CeO_2_ are often reported to exhibit relatively high PZC values [121,122]. ZrO_2_ and TiO_2_ typically exhibit intermediate PZC values [121]. Fe_3_O_4_ also exhibits amphoteric surface behavior [87]. Consequently, inorganic oxide surfaces can exhibit positive or negative surface charge depending on the solution pH relative to the PZC. These pH-dependent surface charge properties contribute to electrostatic interactions with charged biomolecules during adsorption and desorption processes. Notably, PZC values can vary with crystal phase, synthesis route, surface treatment, and measurement conditions; therefore, careful interpretation is required when comparing different materials or studies [121].

Surface hydroxy groups constitute key interfacial functional groups on inorganic oxide surfaces and act as hydrogen-bond donors and acceptors involved in the formation of interfacial hydration structures at oxide–water interfaces [123]. Interfacial water molecules interact with these hydroxyl groups through hydrogen bonding, and the resulting water organization depends on hydroxyl group density, surface termination, and crystal structure [123–126]. ZrO_2_ and TiO_2_ surfaces undergo water-induced hydroxylation under aqueous conditions, where coordinatively unsaturated metal centers with hydroxy groups interact with interfacial water molecules through hydrogen bonding [27,40]. SiO_2_ surfaces present siloxane networks bearing silanol groups, which govern the heterogeneity of hydrogen-bonding sites available for interfacial water [123]. Differences in hydroxy group distribution and surface termination lead to variations in interfacial water structure at oxide–water interfaces, which in turn modulate the hydration-dependent adsorption of biomolecules, including proteins and peptides.

Surface functionalization introduces additional interaction modes beyond intrinsic oxide surface chemistry. Metal-ion immobilization, organic ligand grafting, phosphate group modification, polymer coatings, and biomolecular conjugation enable electrostatic, coordination-mediated, hydrophobic, and affinity-based adsorption mechanisms [19,59,64,66,68,70]. SiO_2_ and Fe_3_O_4_ are widely utilized as functionalization platforms because of their chemical stability, synthetic versatility, and compatibility with diverse surface modification strategies [59,64,66,68,70,81,88]. ZrO_2_ is employed as a platform for phosphate-functionalized surface modification because of the strong affinity between zirconium oxide surface sites and phosphate moieties [19,127]. Such surface engineering approaches expand the applicability of inorganic oxides to selective purification systems for proteins and peptides.

Crystal structure and morphology of inorganic oxides also influence adsorption behavior through their effects on surface-site density, pore accessibility, and interfacial structure, even among chemically identical materials. For example, anatase- and rutile-phase TiO_2_ exhibit different phosphate adsorption characteristics [128]. Particle size (e.g. nanoparticles) and porosity (e.g. mesoporous structures) influence protein adsorption kinetics through differences in accessible surface area and transport properties [129–131]. In Fe_3_O_4_-based systems, the inverse spinel structure provides magnetic responsiveness, enabling rapid separation as an operational advantage [132].

## Material-specific applications in protein and peptide separation

### ZrO_2_

#### Surface chemistry and fundamental binding mechanisms of ZrO_2_

ZrO_2_ surfaces are amphoteric, possessing both acidic and basic sites [26,133,134]. Surface zirconium centers function as Lewis acids that coordinate oxygen donors, whereas surface hydroxyl groups act as Brønsted acid and base sites [26]. The nature and distribution of these surface sites depend strongly on the crystal phase [26]. Among ZrO_2_ polymorphs, the monoclinic form is thermodynamically stable under ambient conditions and exhibits a relatively high surface density of hydroxy groups [135,136]. Because of these characteristics, monoclinic ZrO_2_ is widely used for biomolecule purification and adsorption studies in aqueous media.

The ZrO_2_ surface exhibits versatile interactions due to the coexistence of Lewis- and Brønsted-acidic and basic sites [26,137]. In particular, coordinatively unsaturated zirconium centers (≡Zr^4+^) possess high charge density and strong oxophilicity [127]. A well-characterized example is the interaction with phosphate species: Zr^4+^ centers preferentially coordinate with phosphate oxygen atoms, which act as Lewis bases, thereby forming stable Zr–O–P coordination bonds [127]. This coordination underpins the selective adsorption of phosphorylated peptides [25]. In addition, phosphate groups can be chemically immobilized on the ZrO_2_ surface, introducing additional negatively charged sites [20]. These phosphate functionalities interact electrostatically with basic amino acid residues such as lysine, arginine, and histidine [138]. Such interactions underpin the selective adsorption of proteins containing localized basic regions, thereby extending the applicability of ZrO_2_ materials beyond phosphorylated peptide enrichment.

#### Phosphorylated peptide enrichment using ZrO_2_

Various ZrO_2_ materials, including nanoparticles, mesoporous beads, and microtips, have been developed for phosphorylated peptide enrichment [22–25]. ZrO_2_ is widely employed as a separation medium in phosphoproteomic analysis because of its affinity for phosphate groups [22,24,25]. In a typical workflow, phosphorylated peptide enrichment using ZrO_2_ is performed after proteolytic digestion [24], where peptide mixtures are loaded onto ZrO_2_ particles under acidic conditions. These conditions suppress non-specific interactions of cationic peptides with the particle surfaces while simultaneously promoting selective coordination of phosphorylated moieties to surface Zr^4+^ centers [24]. Non-phosphorylated peptides are removed during washing steps, whereas bound phosphorylated peptides are eluted using phosphate-containing buffers, alkaline solutions, or other competitive ligands that disrupt Zr–O–P interactions [24]. In addition, the use of surface modifiers, such as aliphatic hydroxy acids, as well as phosphonate-functionalized ZrO_2_, effectively reduces non-specific adsorption and improves enrichment efficiency, particularly for multiply phosphorylated peptides [28,29]. These procedures enable reproducible and efficient isolation of phosphorylated peptides from complex peptide mixtures. Because of its high chemical stability, ZrO_2_ can be regenerated and reused even after harsh washing processes [23]. Recent studies have developed hybrid ZrO_2_ materials for efficient phosphorylated peptide enrichment from limited sample amounts [139]. Collectively, these properties—high surface area, tunable selectivity, and chemical robustness—make ZrO_2_ suitable for reproducible and efficient phosphorylated peptide and protein enrichment.

#### Phosphate-modified ZrO_2_ for protein purification

Phosphate-modified ZrO_2_ (ZrO_2_-P) materials have been developed as separation media for protein purification. ZrO_2_-P refers to ZrO_2_ surfaces modified with phosphate- or phosphonate-containing compounds, which are immobilized through Lewis acid–base interactions between phosphate groups and surface zirconium sites [19,20]. Negatively charged phosphate groups immobilized on ZrO_2_ surfaces facilitate protein adsorption through electrostatic interactions with basic amino acid residues [138]. The bound proteins can be eluted by increasing the ionic strength or by mild pH adjustment, allowing purification under relatively gentle conditions [19–21,30,31].

**Figure 1.**
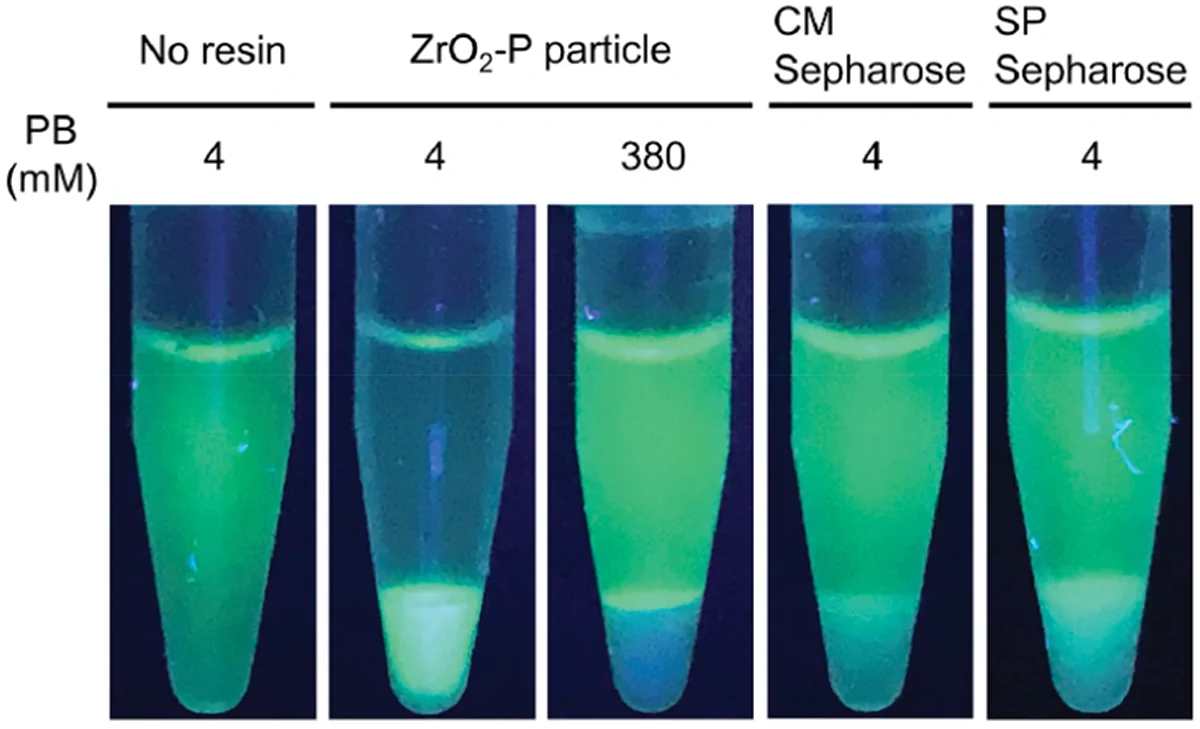
Fluorescence images of GFP–His-expressing lysates after incubation with ZrO_2_-P particles, CM sepharose, or SP sepharose and brief centrifugation. GFP–His localization reflects adsorption to the particles/resins under 4 mM PB (green fluorescence under 254-nm light). Reproduced from Kanoh *et al*., *J. Chromatogr. A*, 1703 (2023) 464,112, with permission from elsevier.

Since proteins containing histidine (His) residues can interact with phosphate groups through electrostatic interactions [138], histidine-rich proteins can be effectively adsorbed onto ZrO_2_-P surfaces. In particular, proteins containing polyhistidine tags (His-tags) exhibit enhanced affinity toward ZrO_2_-P due to multivalent interaction with surface phosphate groups [21]. Such interactions enable the selective capture of recombinant proteins as an alternative to conventional immobilized metal affinity chromatography (IMAC) using heavy metal ions [140]. In comparison, conventional cation-exchange resins exhibit minimal binding of His-tagged proteins under the same conditions, whereas ZrO_2_-P shows strong and controllable adsorption and desorption of these proteins (Figure 1) [21]. Immunoglobulins, which contain localized basic amino-acid-rich regions (e.g. the CL–CH1–hinge region in immunoglobulin G), can also electrostatically interact with surface phosphate groups [138]. These interactions facilitate the adsorption of immunoglobulins onto ZrO_2_-P particles [138]. Based on these adsorption properties, ZrO_2_-P is employed for the purification not only of immunoglobulin G (IgG), which is widely used in therapeutic antibody products [19,31–33], but also of other immunoglobulin classes, such as IgM and IgA, which are being explored for therapeutic and prophylactic applications [20,30,31,34]. In general, affinity protein-based columns, such as Protein A column and Protein L column, are widely used for antibody purification, but these ligands are sensitive to harsh conditions [141,142]. Repeated purification processes can reduce ligand activity [141,142]. Phosphate-functionalized ZrO_2_ particles have the potential to serve as an alternative column filler for an initial enrichment step, allowing antibodies to be captured gently. This pre-purification reduces contaminants and improves the quality of subsequent affinity protein-based purification in terms of antibody purity, yield and activity.

### TiO_2_

#### Surface chemistry and fundamental binding mechanisms of TiO_2_

TiO_2_ surfaces contain both acidic and basic surface sites—under‑coordinated Ti^4+^ cations act as Lewis‑acidic centers, while surface oxygen atoms and hydroxy groups exhibit weak basic character [39,117]. The density and distribution of these acid–base sites can be influenced by factors such as crystal structure and surface hydration [143]. TiO_2_ mainly occurs in anatase and rutile crystal phases, both exposing Ti–O surface terminations but differing in lattice structure and surface hydroxylation behavior [144,145]. Surface hydroxy groups undergo protonation and deprotonation depending on pH, thereby modulating the surface charge [143]; the resulting electrostatic interactions govern protein adsorption behavior and further depend on solution conditions such as ionic strength [146].

TiO_2_ is available in multiple formats, such as anatase nanoparticles, mesoporous beads, and microtips [25,35,38,147]. These formats differ in surface area and accessibility, thereby affecting the capture efficiency of proteins and peptides [36]. Anatase surfaces tend to form abundant hydroxy groups, which can directly coordinate with oxygen-containing ligands such as carboxylate groups [148] and phosphate groups [149]. In particular, Ti–O–P linkages underlie the selective adsorption of phosphorylated peptides and proteins and are widely employed in TiO_2_-based metal oxide affinity chromatography (MOAC) for phosphorylated peptide enrichment [147]. Anatase surfaces exhibit strong affinity for water molecules and form structured interfacial hydration layers under aqueous conditions [40,41]. These hydration layers can modulate the approach and adsorption of amino acids to the surface [150,151]. Variations in solution conditions alter the stability and organization of these hydration layers, which can be exploited to modulate protein–surface interactions [37].

#### Enrichment of phosphorylated peptide using TiO_2_

**Figure 2.**
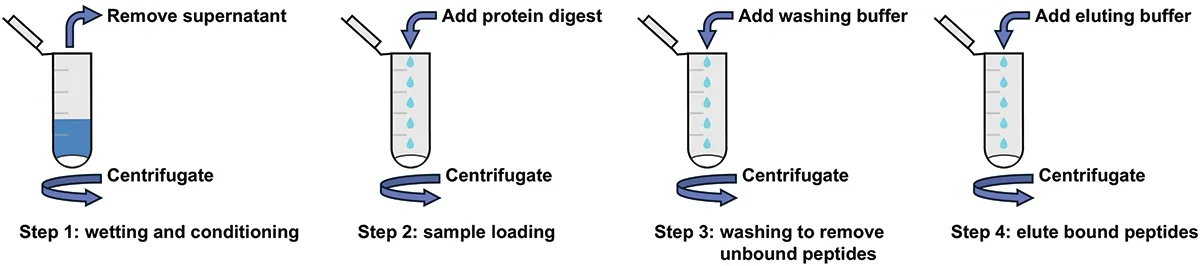
Schematic workflow of single-tube phosphorylated peptide enrichment using metal oxide particles. The procedure involves (1) wetting and conditioning of the particles, (2) sample loading, (3) washing to remove unbound peptides, and (4) elution of bound phosphorylated peptides. This batch-wise approach enables simple, scalable, and controllable enrichment suitable for downstream proteomic analysis. Adapted from Pocsfalvi, G., *Methods in Enzymology*, 457 (2009) 81–96, with permission from elsevier.

TiO_2_-based phosphorylated peptide enrichment is widely used in phosphoproteomic workflows [35,147,152,153]. This material is chemically stable and maintains consistent performance across repeated experiments, ensuring reliable recovery of phosphorylated peptides from complex biological samples [42]. In addition, enrichment can be performed in a single tube, providing a simple and convenient workflow suitable for high-throughput applications (Figure 2) [43,154]. The enrichment process is typically performed after proteolytic digestion [152], where phosphorylated peptides are loaded under acidic conditions to suppress non‑specific binding, thereby promoting specific phosphate-TiO_2_ interactions [16]. For example, organic acids such as 2,5-dihydroxybenzoic acid (DHB) are used to acidify the solution, thereby enhancing phosphate-specific binding to TiO_2_ [16,44]. Bound phosphorylated peptides are eluted from TiO_2_ using phosphate-containing buffers or alkaline solutions (e.g. NH_4_OH at pH 10.5) without degrading phosphorylation modifications [152]. Additives such as glycerol, secondary amines, and bis‑Tris propane improve phosphorylated peptide recovery, particularly for hydrophilic, acidic, and longer peptides [45]. Specifically, glycerol enhances selectivity for singly phosphorylated peptides, whereas secondary amines and bis‑Tris propane broaden the applicability of TiO_2_ to peptides with diverse lengths and compositions [45]. In addition, amine functionalization of TiO_2_ surfaces can tune surface Lewis acidity and hydrophilicity, thereby enhancing selectivity for phosphorylated peptides [35]. Other surface modifiers, such as aliphatic hydroxy acids and phosphonates, can reduce non-specific adsorption and improve enrichment efficiency, particularly for multiply phosphorylated peptides [28,29]. Such optimization of solution conditions enhances overall recovery efficiency and supports robust downstream mass spectrometric analysis.

#### Protein purification via interfacial water on TiO_2_

Interfacial water at anatase surfaces is strongly adsorbed due to abundant surface hydroxy groups [40,41]. Water molecules in the vicinity of the TiO_2_ surface form structured hydration layers that mediate hydrogen bonding and solvent organization, thereby modulating solute–surface interactions [150,151]. This interfacial phenomenon can be exploited for the selective separation of proteins, even those with similar sizes and net charges [37]. The introduction of ionic liquids on TiO_2_ creates a heterogeneous local environment composed of cations and anions [37]. This environment alters the structure of interfacial water and, in turn, modulates protein–surface interaction [37]. As a result, the hydration environment on the TiO_2_ surface in the presence of ionic liquids differs from that in their absence, leading to distinct adsorption and retention behaviors. This approach enables selective separation while preserving the intrinsic surface chemistry and stability of TiO_2_.

### SiO_2_

#### Surface chemistry and fundamental binding mechanisms of SiO_2_

Amorphous SiO_2_ presents a hydroxylated surface composed of silanol groups (≡Si–OH) and siloxane bridges (≡Si–O–Si≡) [155]. Since surface silanol groups exhibit amphoteric behavior depending on their protonation state [62], they can participate in hydrogen bonding and electrostatic interactions with proteins and peptides [156]. In particular, deprotonation of silanol groups generates negatively charged ≡Si–O^−^ sites under neutral to alkaline aqueous conditions, enabling electrostatic interactions [157]. In contrast, siloxane bridges (≡Si–O–Si≡) are generally chemically inert under typical conditions [158].

Amorphous SiO_2_ is widely used in diverse separation systems due to its high specific surface area and high silanol density [159,160]. Porous structures enhance accessibility to internal surface area, facilitating efficient interactions with biomolecules [159]. Owing to these characteristics, SiO_2_ is widely used as stationary phase for protein and peptide separation. The negatively charged SiO_2_ surface interacts electrostatically with positively charged amino acid residues, such as lysine [161]. Therefore, proteins with high isoelectric points exhibit strong adsorption under suitable pH conditions, whereas proteins with lower isoelectric points remain weakly bound [46]. Although protein adsorption on SiO_2_ surfaces is predominantly governed by electrostatic interactions, other interactions, such as hydrophobic interactions, also contribute [17,162]. pH and ionic strength modulate these electrostatic interactions, thereby controlling adsorption and desorption behavior and improving adsorption selectivity for protein purification.

SiO_2_ materials also exist in crystalline polymorphic forms. α-quartz is a representative crystalline form of SiO_2_ that shares the Si–O tetrahedral framework of amorphous SiO_2_ but differs in surface silanol density and spatial arrangement. Compared with amorphous SiO_2_, α-quartz exhibits a more ordered arrangement of surface oxygen atoms and distinct surface silanol configurations [122,163]. These characteristics can be exploited in tag-based purification systems (e.g. Si-tag and CotB1-tag), where selective adsorption of engineered peptide sequences is achieved through controlled SiO_2_–protein interfacial interactions [50–53].

Surface silanol groups serve as reactive sites for chemical functionalization, such as covalent coupling [162,164]. Organosilane reagents, which react with ≡Si–OH groups, form stable Si–O–Si–R linkages, thereby introducing functional groups onto the SiO_2_ surface. The functional moieties include metal-chelating ligands and organic small molecules [54,59]. Such functionalization enables diverse interaction modes, including electrostatic attraction, coordination bonding, and hydrophobic interaction [59,64,66]. Additional strategies, such as polymer coating and ligand immobilization, can further enhance protein and peptide selectivity by providing denser or more specific binding sites [68,70]. Collectively, these approaches establish SiO_2_ as a versatile platform for constructing tailored interfaces in protein and peptide separation systems.

#### Purification of proteins using bare SiO_2_

Bare SiO_2_ particles enable protein purification through electrostatic and hydrophobic interactions [17,46]. Industrial-scale studies using preparative SiO_2_ gels have demonstrated the effectiveness of SiO_2_ columns for the purification of recombinant proteins [17]. Notably, single-step purification on SiO_2_ can replace conventional multi-step processes, including hydrophobic interaction chromatography (HIC), ultrafiltration/diafiltration (UF/DF), and cation-exchange (CEX) chromatography [17]. Bare SiO_2_ particles also exhibit pH-dependent competitive adsorption of proteins with different isoelectric points, enabling the fractionation of proteins such as lysozyme, myoglobin, and cytochrome c [46]. This approach provides a cost-effective and high-performance purification platform, achieving high purity and recovery while reducing process complexity.

Bare SiO_2_ has also been applied to the purification of fusion-tagged proteins because it interacts specifically with certain peptide tags. For example, the Si‑tag, derived from bacterial ribosomal protein L2, binds tightly to SiO_2_ surfaces. Si-tagged proteins bound to these surfaces can be eluted by adding divalent cations [50,51]. Notably, the interaction between the Si-tag and SiO_2_ surfaces remains effective even in the presence of protein denaturants, allowing Si-tagged proteins to be purified using SiO_2_ even if they are expressed as insoluble aggregates [50]. Cationic residue-rich tags (such as the CotB1p tag and (RH)4 tag) mediate selective adsorption through electrostatic interactions between their positively charged arginine residues and the negatively charged SiO_2_ surface [52,74]. The Car9 tag, originally identified as a peptide that bind to carbonaceous materials, also shows strong affinity for SiO_2_ [47,75]. Car9-tagged proteins can be purified under mild conditions with moderate pH shifts and the addition of lysine [47,75]. Bare SiO_2_ can also be used for the purification of His-tagged proteins, which are widely employed in recombinant protein production, with elution achieved by the addition of lysine and arginine [53]. These properties of bare SiO_2_ thus provide a versatile platform for both tag-free and tag-mediated protein purification workflows.

#### Enrichment of phosphorylated peptides and purification of proteins using functionalized SiO_2_

Surface modification expands the functionality of SiO_2_, enabling selective protein purification based on tailored interfacial interactions. Chemical modification introduces defined binding motifs and converts SiO_2_ into an affinity platform. Representative strategies include the immobilization of metal ions, the introduction of organic small molecules, coating with functional polymers, and the immobilization of proteins and biomolecular ligands.

Immobilization of metal ions on SiO_2_ enables coordination-driven purification through metal–ligand interactions. Chelating ligands, such as iminodiacetic acid (IDA) and nitrilotriacetic acid (NTA), covalently introduced onto SiO_2_ surfaces can coordinate transition metal ions including Cu^2+^ and Co^2+^ [59,63]. The surface-bound metal centers further coordinate with electron-donor groups, particularly histidine residues, thereby enabling IMAC-based purification [59]. For example, Co^2+^-modified SiO_2_ particles are used for the purification of His-tagged proteins under mild conditions [59]. Cu^2+^-modified SiO_2_ capillaries exhibit selective retention of certain proteins via coordination bond to Cu^2+^ centers [63]. Metal oxide coatings further extend this coordination framework. Specifically, ZrO_2_- and TiO_2_-coated SiO_2_ surfaces present Lewis-acidic metal sites that strongly interact with phosphate groups, allowing the selective enrichment of phosphorylated peptides [48]. These inorganic modification strategies are widely applicable to tag-based and post-translational modification-specific protein purification [48,59,63].

Organic small molecule functionalization offers defined interaction motifs for protein purification. Tetrazole- and morpholine-modified SiO_2_ can function as cation- or anion-exchange media for protein purification [64,65]. Cholesterol- and octadecylsilyl (C18)‑bonded SiO_2_ materials exhibit hydrophobicity and hence are used in reversed‑phase chromatography for protein separation [55,66]. In particular, cholesterol-bonded SiO_2_ has also been used for protein purification coupled with protein refolding because of its moderate hydrophobicity [55]. Dye molecules that mimic enzyme cofactors and substrates offer selective binding to corresponding enzymes. Cibacron Blue F3G-A immobilized on SiO_2_ acts as a pseudo-affinity ligand [54]. Because this dye interacts with nucleotide-binding proteins, the functionalized SiO_2_ can be used for affinity purification of enzymes such as dehydrogenases [54]. Other small molecules immobilized on SiO_2_ surfaces also provide different selectivities. For example, glutathione‑modified SiO_2_ selectively enriches N‑linked glycopeptides through hydrophilic interactions, thereby enabling the purification of GST-tagged proteins [49,56]. Boronic acid immobilized on SiO_2_ forms reversible covalent interactions with cis-diol groups, which can be applied to the purification of glycoproteins [67].

Polymer‑modified SiO_2_ provides high ligand density and multivalent interactions, leading to high binding capacity and separation efficiency. Cationic polymers introduce high‑density positive charges to the SiO_2_ surfaces, functioning as anion‑exchangers in protein purification [68]. In contrast, anionic polymers act as cation‑exchangers [69]. Polymer modification can thus control interaction strength, selectivity, and binding capacity in protein purification.

**Figure 3.**
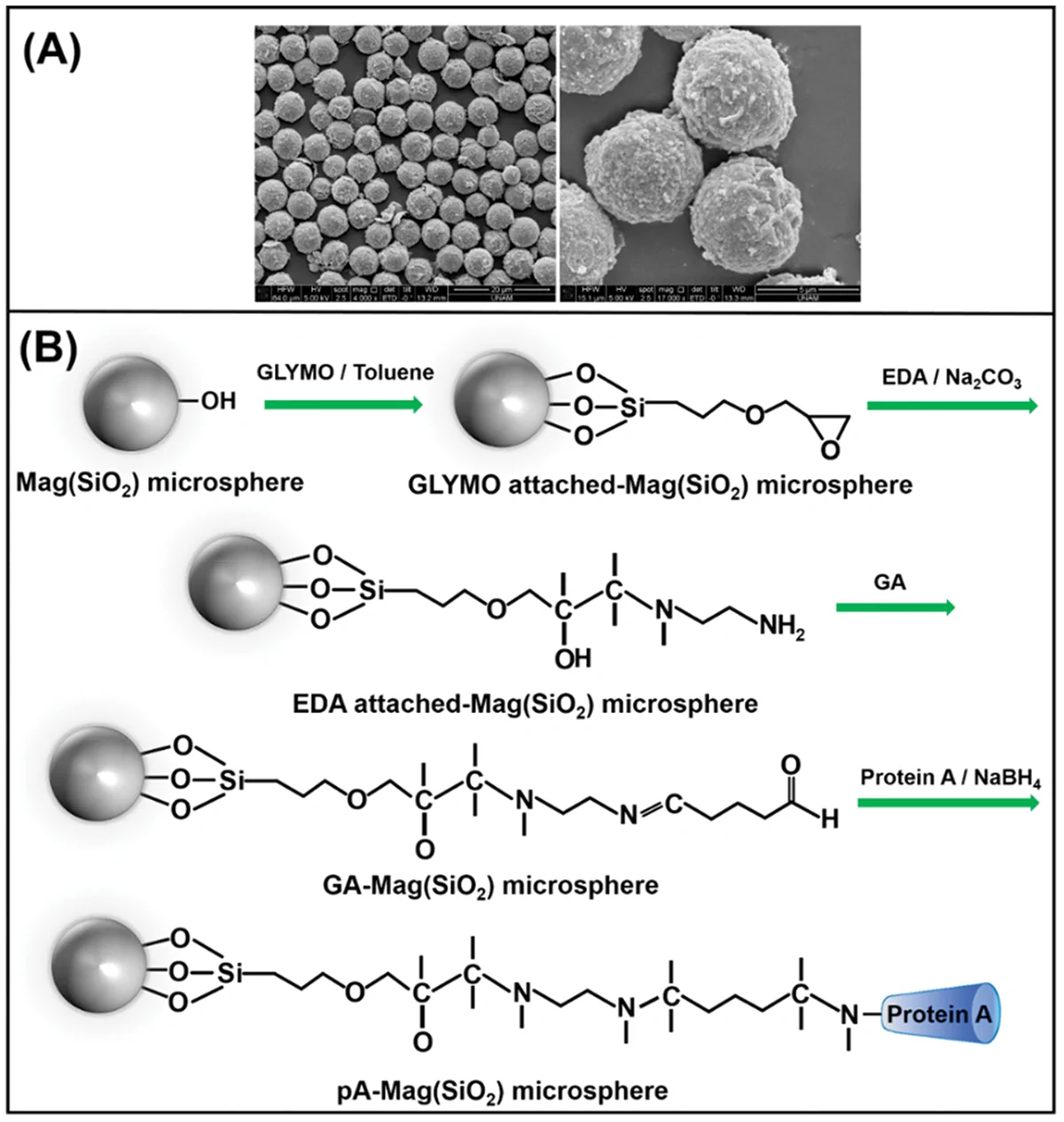
Schematic illustration of protein a immobilization on magnetic porous silica (Mag(SiO_2_)) microspheres. Surface hydroxyl groups of the silica allow covalent attachment of protein A, enabling selective capture of immunoglobulin G. Reproduced from Salimi et al., *Int. J. Biol. Macromol*., 111 (2018) 178–185, with permission from Elsevier.

Immobilization of specific proteins and peptides on SiO_2_ offers highly specific affinity interactions. For example, magnetic porous SiO_2_ (Mag(SiO_2_)) microspheres can be functionalized with protein A and enable the selective capture of immunoglobulin G (Figure 3) [57]. Protein A immobilized on mesoporous SiO_2_ selectively binds to immunoglobulin G, allowing selective antibody purification [70]. Lectins such as concanavalin A immobilized on SiO_2_ recognize glycan structures, enabling the enrichment of glycoproteins and glycopeptides [71]. Affinity peptides can also be introduced onto SiO_2_ surfaces to purify target antibody through specific peptide–protein interactions [72]. These protein- and peptide-based ligand strategies offer particularly high specificity among the various SiO_2_ modification strategies and are suitable for the purification of structurally defined proteins.

### Fe_3_O_4_

#### Surface chemistry and fundamental binding mechanisms of Fe_3_O_4_

Fe_3_O_4_ surfaces have both acidic and basic functionalities [87]. Coordinatively unsaturated Fe sites on the surfaces act as Lewis-acidic centers for coordinating oxygen-donor ligands [86,87,118]. Surface hydroxy groups, formed spontaneously in aqueous systems, contribute to the acid–base character through protonation and deprotonation depending on pH [87]. Fe_3_O_4_ surface acid–base site density also varies with crystal phase [87]. These structural and chemical properties render Fe_3_O_4_ suitable for selective adsorption of proteins and peptides.

Fe_3_O_4_ surfaces have coordination bonds of oxygen- and nitrogen-donor groups of biomolecules [165–168]. Typically, oxygen-donor groups, such as carboxylates and phosphates, coordinate to Fe centers [167,168]. Phosphate groups form particularly stable multidentate Fe–O–P linkages [167]. Nitrogen atoms of imidazole groups, as in histidine residues, can also coordinate to Fe sites [166]. Such Fe–ligand coordination, especially with phosphate and imidazole groups, together with electrostatic interactions, governs the selective adsorption of proteins and phosphorylated biomolecules.

γ-Fe_3_O_4_ nanoparticles exhibit ferrimagnetism because of their inverse spinel crystal structure [169,170]. In this structure, Fe^2+^ and Fe^3+^ ions occupy tetrahedral and octahedral sites with antiparallel spin alignment [169,170], producing a net magnetic moment and hence exhibiting characteristic magnetization [169,170]. Strong magnetization combined with nanoscale size allows the particles to respond quickly to external magnetic fields. These intrinsic magnetic features can be used for the magnetic separation and purification of proteins and peptides bound to the nanoparticles.

#### Enrichment of phosphorylated peptide and purification of protein using bare Fe_3_O_4_

Bare Fe_3_O_4_ nanoparticles exhibit selective interactions with phosphorylated peptides and His-tagged proteins [18,76,77]. The formation of Fe–O–P linkages enables preferential binding of phosphorylated peptides over non-phosphorylated counterparts [76]. This binding can be optimized by pH and buffer composition, thereby enhancing both purity and recovery [76]. Additionally, the magnetic properties of Fe_3_O_4_ allow rapid separation of the enriched fraction from complex mixtures [76]. Together, these features provide a simple, ligand-free approach for phosphorylated peptide enrichment prior to mass spectrometric analysis.

Bare Fe_3_O_4_ nanoparticles can also capture His-tagged recombinant proteins directly from crude lysates [18]. The imidazole groups of histidine residues coordinate with metal ions, similar to immobilized metal ion affinity chromatography (IMAC) [171]. Proteins lacking His-tags exhibit minimal binding, whereas His-tagged proteins are selectively adsorbed onto Fe_3_O_4_ surfaces and subsequently eluted using imidazole-containing buffers [18]. In addition, peptide tags such as His-tag, CotB1p-tag, and Car9-tag, which have affinity for both Fe_3_O_4_ and SiO_2_, enable sequential workflows that involve magnetic capture followed by purification using SiO_2_ surfaces, or vice versa [77].

#### Enrichment of phosphorylated peptides and purification of protein using modified and coated Fe_3_O_4_

Surface modification and coating enhance the specificity of Fe_3_O_4_ nanoparticles toward peptides and proteins. Various functional groups, including chelating ligands and boronic acids, provide selective binding sites for target proteins [78–82,88]. Inorganic coatings further confer affinity for proteins and peptides while preserving the magnetic core [81,83–85].

**Figure 4.**
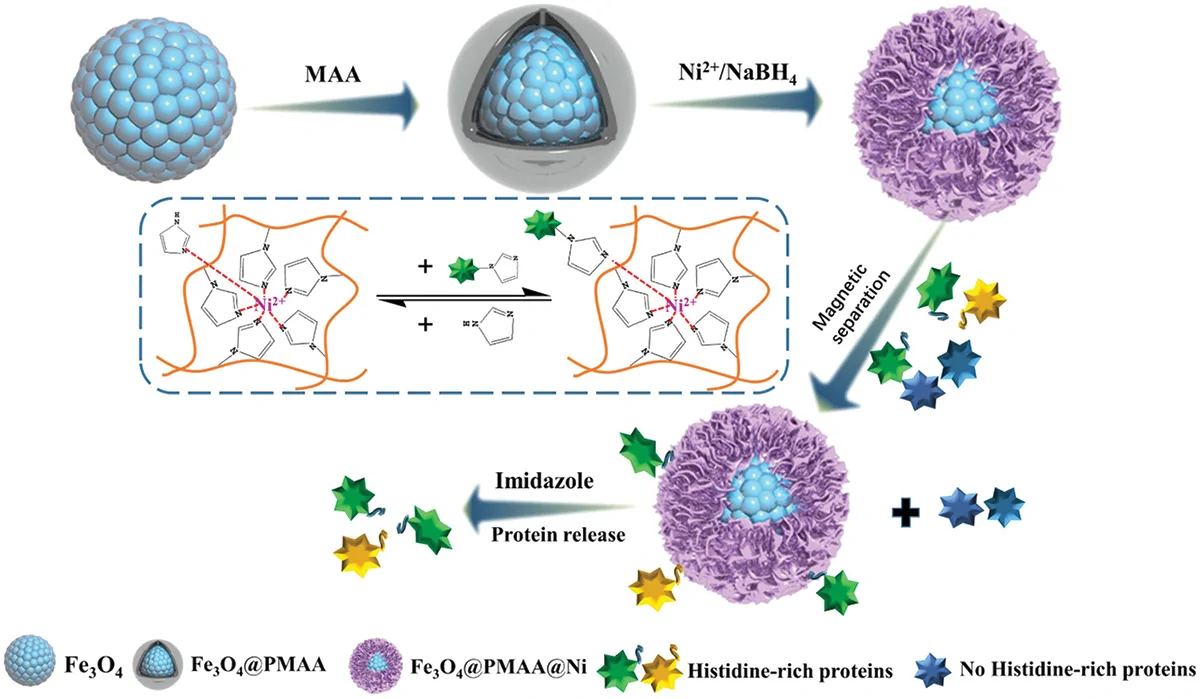
Preparation of core–shell Fe_3_O_4_@PMAA@Ni microspheres and their application for efficiently and selectively separating His-rich proteins. Reproduced from Wang et al., *ACS Appl. Mater. Interfaces*, 13 (2021) 11,166–11,176, with permission from ACS.

Ni^2+^ ions immobilized via polymer layers on Fe_3_O_4_ nanoparticles provide selective binding sites for His-tagged proteins and His-rich proteins [78,82,88]. A core–shell architecture of Fe_3_O_4_@PMAA@Ni microspheres was developed along with a workflow for magnetic capture and selective enrichment of His-rich proteins (Figure 4) [82]. Boronic acid moieties introduced on the Fe_3_O_4_ surface capture glycopeptides and glycoproteins through cis-diol interactions [79,81]. Immobilization of protein A on Fe_3_O_4_ affords high-affinity binding to antibody Fc regions [80]. The resulting Fe_3_O_4_ nanoparticles retain magnetic separability, supporting efficient enrichment workflows.

Inorganic materials such as SiO_2_, ZrO_2_, and TiO_2_ are used to form core–shell structures with Fe_3_O_4_ cores [81,83–85]. In particular, SiO_2_-coated magnetic nanoparticles can be functionalized with boronic acid ligands for glycopeptide and glycoprotein enrichment and with proteolytic enzymes such as trypsin for on-particle digestion [81,89]. ZrO_2_ and TiO_2_ coating layers on Fe_3_O_4_ exhibit affinity for phosphorylated species [83,84]. These materials enable the enrichment of phosphorylated peptides from complex peptide mixtures through magnetic separation.

More recently, aptamer-functionalized Fe_3_O_4_ nanoparticles have been developed for the selective capture of extracellular vesicles, extending magnetic enrichment strategies from protein- and peptide-level targets to nanoscale biological assemblies through sequence-specific molecular recognition [172]. These advances suggest that Fe_3_O_4_-based platforms can serve as versatile tools for multiscale biological separations and integrated bioanalytical workflows.

### Other materials

#### Al_2_O_3_

Al_2_O_3_ surfaces exhibit both acidic and basic sites [92]. Surface aluminum centers (Al^3+^) function as Lewis acids, and surface hydroxy groups can undergo reversible protonation and deprotonation [92,93]. The density and distribution of these sites depend on crystal structure and can be further controlled by surface hydroxylation and preparation conditions such as calcination temperature and pretreatment conditions [173]. For example, γ-Al_2_O_3_ exhibits a greater density of coordinatively unsaturated Al^3+^ sites compared with other common polymorphs such as α- and θ-Al_2_O_3_ [173–176]. These Al^3+^ sites can bind oxygen-donor groups, such as carboxylate and phosphate moieties, through coordination bonds [177,178]. Such binding is also influenced by pH-dependent protonation of surface hydroxy groups [177,178]. These physicochemical characteristics govern the adsorption behavior of peptides and proteins on Al_2_O_3_.

The combination of Lewis-acidic Al^3+^ sites and modifiable surface hydroxy groups enables Al_2_O_3_ to exhibit electrostatic interactions [177]. In fact, unmodified γ-Al_2_O_3_ has been used in high-performance liquid chromatography (HPLC) to separate proteins through these electrostatic interactions, where the retention depends on the difference between a protein’s isoelectric point (p*I*) and the point of zero charge (PZC) of γ-Al_2_O_3_ [90]. Specifically, proteins carrying charges opposite to the surface are preferentially retained. Modulating pH and ionic strength allows fine-tuning of both retention and selectivity [90]. In addition, Lewis-acidic Al^3+^ sites serve as specific coordination sites for phosphate groups, enabling selective capture of phosphorylated peptides [91]. This mechanism has been exploited in Al_2_O_3_-coated magnetic core–shell microspheres, which allow rapid and highly specific enrichment of phosphorylated peptides [91]. Compared with commercial TiO_2_-based materials, Al_2_O_3_-coated systems exhibit higher selectivity while minimizing non-specific adsorption [91].

Surface modification of Al_2_O_3_ is effective for protein separation based on differences in hydrophobicity. Octadecyl- and polybutadiene-bonded Al_2_O_3_ are used in reversed-phase HPLC of proteins and peptides while maintaining the structural advantages of the Al_2_O_3_ support [94,95].

#### ZnO

ZnO surfaces are amphoteric and contain Lewis acidic zinc sites (Zn^2+^) [101,119]. The surface charge of ZnO can thus be controlled by pH, affecting biomolecule adsorption [97,101].

ZnO surfaces adsorb proteins, including bovine serum albumin (BSA), lysozyme, and cytochrome c [97–99], and adsorption behavior depends on pH [99]. Electrostatic interactions predominantly contribute to adsorption, while hydrogen bonding and van der Waals interactions may also be involved [96,100]. Although ZnO has primarily been studied for protein adsorption, it also holds potential as a material for protein and peptide purification with appropriate control of solution conditions.

#### CeO_2_

CeO_2_ exhibits amphoteric surface properties and contains Lewis-acidic cerium sites (Ce^4+^) [104–106]. Phosphate and carboxylate groups adsorb onto CeO_2_ surfaces; this adsorption is influenced by surface properties such as the density of hydroxy groups and the strength of Lewis acidity [179–182]. These properties of CeO_2_ are applied to the enrichment and isolation of phosphorylated peptides and proteins [102,103]. In addition, CeO_2_ microspheres exhibit peroxidase-like activity and have been proposed as nanozyme platforms for the colorimetric quantification of phosphoproteins in complex samples [102].

CeO_2_ surfaces can also be used for adsorption of non-phosphorylated proteins. Some proteins, including bovine serum albumin (BSA) and lysozyme, are reported to adsorb onto CeO_2_ nanoparticles [97,98,183]. The adsorption can be controlled by changing the solution pH [183,184]. Proteins may undergo local conformational changes upon binding to CeO_2_ in some cases [97,98]. Although CeO_2_ has not been systematically studied for protein purification, its pH-dependent adsorption may be leveraged for this purpose, provided that conditions are carefully controlled to minimize protein denaturation.

#### SnO_2_, Nb_2_O_5_, Ta_2_O_5,_ Ga_2_O_3_

SnO_2_, Nb_2_O_5_, Ta_2_O_5_, and Ga_2_O_3_ contain Lewis-acidic metal centers (Sn^4+^, Nb^5+^, Ta^5+^, Ga^3+^) [109,111,113,115,116,120]. These centers coordinate with phosphoryl oxygen atoms. SnO_2_ and Nb_2_O_5_ act as affinity materials for phosphorylated peptides and are employed in proteomic analysis [107,108,110]. Phosphorylated peptides retained on the oxide surfaces under acidic conditions are eluted by a drastic change in pH or by the introduction of phosphate-containing buffers [107,108,110]. SnO_2_ showed phosphorylated peptide enrichment performance comparable to that of TiO_2_ and enabled selective adsorption of phosphorylated peptides under simple solvent conditions [107]. In some cases, SnO_2_ also provided higher recovery of singly phosphorylated peptides than TiO_2_ [108]. Nb_2_O_5_ exhibits selectivity toward phosphorylated peptides that differed from that of TiO_2_, suggesting its potential complementary use for phosphorylated peptide enrichment [110]. Similar phosphorylated peptide adsorption behavior is observed for Ta_2_O_5_- and Ga_2_O_3_-coated oxide materials on magnetic nanoparticles [112,114].

## Comparative discussion and integration

Metal oxides exhibit differences in Lewis acidity, amphotericity, and hydroxy group density, which influence their interactions with proteins and peptides. ZrO_2_ and TiO_2_ possess high-valent metal centers (Zr^4+^ and Ti^4+^) that form coordination bonds with oxygen-donor groups such as phosphate groups [127,149]. Fe_3_O_4_ contains moderate Lewis-acidic Fe^2+^/Fe^3+^ sites, exhibiting pH-dependent surface charge and magnetic separability [118,169,170]. SiO_2_ exhibits negligible intrinsic Lewis acidity; instead, its surface silanol groups mediate electrostatic interactions. Across these inorganic oxides, crystal phase and hydroxylation affect site accessibility, governing the balance among coordination bonds, electrostatic interaction, and hydration-mediated interaction.

ZrO_2_ and TiO_2_ are among the most widely used materials for phosphorylated peptide enrichment, as they bind phosphate groups via surface Lewis acid sites [24,152]. ZrO_2_ preferentially captures singly phosphorylated peptides rather than multiply phosphorylated peptides [25]. In contrast, TiO_2_ exhibits particularly high selectivity for multiply phosphorylated peptides compared with singly phosphorylated ones [25]. The complementary selectivities of ZrO_2_ and TiO_2_ can be exploited in combined workflows to broaden the range of phosphorylated peptides recovered [25,185]. Various materials, such as Fe_3_O_4_, Al_2_O_3_, SnO_2_, Nb_2_O_5_, Ta_2_O_5_, CeO_2_, and Ga_2_O_3_ have also been reported to enrich phosphorylated peptides [91,102,103,107,108,110,112,114].

Affinity purification of tagged proteins is essential for high-purity purification of recombinant proteins. Typically, His‑tagged proteins can be selectively captured on inorganic and hybrid materials, offering distinct advantages. Phosphate-functionalized ZrO_2_ particles enable purification of His-tagged proteins under near-neutral pH by adjusting phosphate concentration, providing a mild and efficient workflow [21]. SiO_2_ with surface silanol groups is also used for His-tagged protein purification. A remarkable advantage of these materials is that they do not contain toxic transition metal ions [53]. An additional advantage of SiO_2_ is its applicability to other tags, including Si-tag, Car9-tag, CotB1p-tag, and GST-tag, demonstrating its versatility [47,50–52,56,74]. Magnetic iron oxide (Fe_3_O_4_) nanoparticles also capture His‑tagged proteins [18]. Both SiO_2_ and Fe_3_O_4_ surfaces can be further modified with chelating agents such as nitrilotriacetic acid (NTA) loaded with Co^2+^, Cu^2+^, or Ni^2+^ to perform immobilized metal affinity chromatography (IMAC) [59,63,78,88]. This modification provides tunable selectivity, reduces non-specific binding, and, particularly for Fe_3_O_4_, combines the advantages of magnetic handling. Dual-affinity strategies using both SiO_2_ and Fe_3_O_4_ surfaces have been developed for multi-step workflows [77]. These strategies enable sequential capture, separation, and elution of recombinant proteins [77]. Taken together, inorganic oxides provide complementary options for tagged protein purification under mild conditions. They also have the potential to adapt to other tag systems through tailored surface modifications.

## Outlook and future perspectives

**Figure 5.**
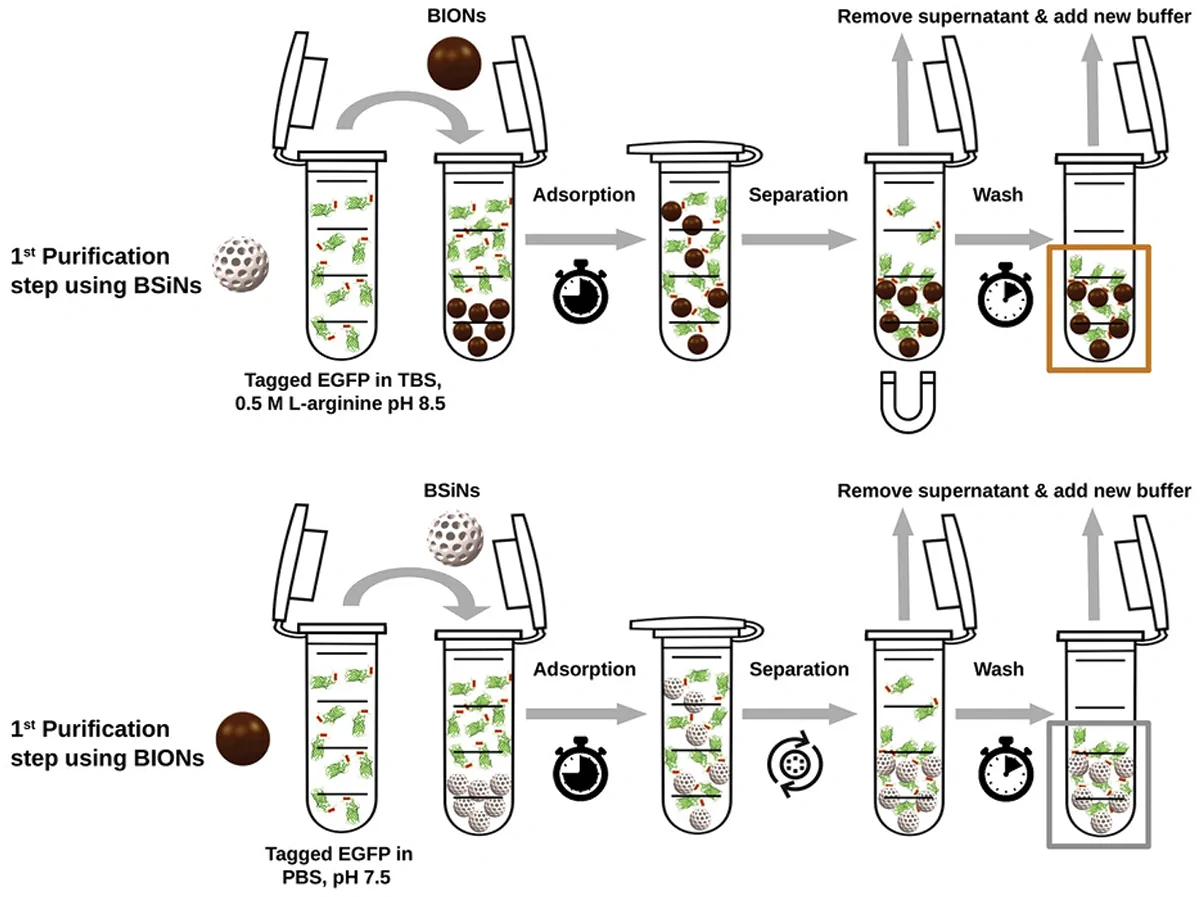
Workflow of affinity-tagged proteins using Fe_3_O_4_ (BIONs) and SiO_2_ (BSiNs) particles. Reproduced from Aguiar and Domingues, *Biotechnol. J*., 18 (2023) 2,300,152, with permission from Wiley.

Inorganic oxides and their hybrid materials offer diverse strategies for protein and peptide purification. The surface chemistry of these materials modulates their interaction modes, influencing selectivity and efficiency. Optimizing workflows requires the careful choice and combination of these materials based on their complementary properties. For example, SiO_2_ and Fe_3_O_4_ support affinity purification of tagged proteins, whereas ZrO_2_ and TiO_2_ enable selective phosphorylated peptides enrichment. Dual-affinity peptide tags, including Car9-derived sequences, further allow protein capture on both SiO_2_ and Fe_3_O_4_, demonstrating the feasibility of integrated workflows (Figure 5) [77]. Li et al. demonstrated that Fe_3_O_4_@TiO_2_–ZrO_2_ core–shell microspheres combine the magnetic separation capability of Fe_3_O_4_ with the distinct phosphate affinities of ZrO_2_ and TiO_2_ to efficiently enrich phosphorylated peptides from complex mixtures [185]. These examples demonstrate that combining inorganic oxides enables more efficient and versatile purification than single-material approaches. For instance, ZrO_2_ enables ligand-free antibody capture under mild conditions for initial sample cleanup [19], whereas SiO_2_ and Fe_3_O_4_ functionalized with affinity ligands provide high selectivity for subsequent polishing steps [70,80]. This sequential use exploits complementary properties, including ligand-free capture, magnetic separation, and surface modification versatility, to improve purification performance. Thus, the combination of inorganic oxides based on their complementary properties enables flexible workflows tailored to specific purification requirements, leading to shorter processing times, higher recovery, and greater reusability.

A understanding of protein–material and peptide–material interactions remain essential. For example, phosphorylated peptides enrichment has been widely examined using inorganic oxides, including ZrO_2_, TiO_2_, SiO_2_, Fe_3_O_4_, Al_2_O_3_, ZnO, CeO_2_, SnO_2_, Nb_2_O_5_, Ta_2_O_5_, and Ga_2_O_3_, each showing distinct selectivity and binding behavior; however, a systematic framework that links surface chemistry, site accessibility, and solution conditions to enrichment performance is still lacking, as direct comparisons across materials remain limited. Establishing standardized comparative evaluation methods and design principles would deepen the understanding of the underlying fundamental interaction mechanisms, thereby contributing to the rational development of efficient, reproducible, and versatile purification workflows.

Novel purification strategies continue to emerge, including new surface engineering approaches. SiO_2_ phases modified with *N*-methylimidazolium ionic liquids provide multi-interaction surfaces that enhance selectivity and resolution for acidic proteins under combined reversed-phase and ion-exchange conditions [186]. Macroporous SiO_2_ functionalized with imidazole-octyl ligands achieves selective isolation of proteins such as ovomucoid and ovotransferrin through electrostatic and hydrophobic interactions [187]. In addition, thermoresponsive polymer interfaces have been developed for temperature-triggered control of protein purification. By reversibly altering surface hydrophilicity and electrostatic properties in response to temperature changes, these systems regulate protein–surface interactions and enable controllable adsorption and desorption under mild conditions [188]. Further exploration of functional polymers, chelating groups, and inorganic oxides for surface modification will be essential, as this will promote the development of versatile purification strategies that expand the range of target proteins while achieving high yield, high purity, and preserved activity.

## Conclusion

Inorganic oxides provide versatile platforms for protein and peptide separation and purification due to their distinct surface chemistry and interaction modes. ZrO_2_ and TiO_2_ efficiently enrich phosphorylated peptides through surface Lewis-acidic sites, with ZrO_2_ favoring singly phosphorylated sequences and TiO_2_ favoring multiply phosphorylated sequences. Phosphate-functionalized ZrO_2_ can be used for antibody pre-enrichment prior to subsequent purification steps. SiO_2_, with both metal-free and metal-ion-mediated affinity, can be tailored with ligands for antibodies, His-tagged proteins, and other fusion proteins. Fe_3_O_4_, which enables rapid magnetic separation, can be used for IMAC-like purification. Combining these materials in multi-step workflows exploits their complementary properties and enables high-purity recovery and efficient fractionation. Expanding the evaluation of emerging oxides, such as Al_2_O_3_, CeO_2_, SnO_2_, Ta_2_O_5_, Nb_2_O_5_, and Ga_2_O_3_ could further enhance selective enrichment strategies. These approaches provide flexible and effective solutions for phosphorylated peptide enrichment, tagged protein purification, and broader applications in proteomics and biopharmaceutical research.

## References

[1] Du M, Hou Z, Liu L, et al. 1Progress, applications, challenges and prospects of protein purification technology. Front Bioeng Biotechnol. 2022;10. doi: 10.3389/fbioe.2022.1028691 PMC9763899 36561042

[2] Sánchez-Trasviña C, Flores-Gatica M, Enriquez-Ochoa D, et al. Purification of modified therapeutic proteins available on the market: an analysis of chromatography-based strategies. Front Bioeng Biotechnol. 2021;9. doi: 10.3389/fbioe.2021.717326 PMC8417561 34490225

[3] Kumar V, Barwal A, Sharma N, et al. Therapeutic proteins: developments, progress, challenges, and future perspectives. 3 Biotech. 2024;14(4):112. doi: 10.1007/s13205-024-03958-z PMC10948735 38510462

[4] Zhu MM, Mollet M, Hubert RS, et al. Industrial production of therapeutic proteins: cell lines, cell culture, and purification. In: Kent JA, Bommaraju TV, Barnicki SD, editors. Handbook of industrial chemistry and biotechnology. Cham, Switzerland: Springer International Publishing; 2017. p. 1639–20. doi: 10.1007/978-3-319-52287-6_29

[5] Tang S, Tao J, Li Y. Challenges and solutions for the downstream purification of therapeutic proteins. Antib Ther. 2024;7(1):1–12. doi: 10.1093/abt/tbad028 38235378 PMC10791043

[6] Cuatrecasas P. Agarose derivatives for purification of protein by affinity chromatography. Nature. 1970;228(5278):1327–1328. doi: 10.1038/2281327a0 5488110

[7] Coskun O. Separation techniques: chromatography. North Clin Istanb. Published online 2016. N Clin Istanb. 2016. doi: 10.14744/nci.2016.32757 PMC5206469 28058406

[8] Nweke MC, McCartney RG, Bracewell DG. mechanical characterisation of agarose-based chromatography resins for biopharmaceutical manufacture. J Chromatogr A. 2017;1530:129–137. doi: 10.1016/j.chroma.2017.11.038 29162233

[9] Pires MC, Jungbauer A, Lau D, et al. Column ageing of agarose-based chromatography in industrial lactoferrin purification. J Chromatogr A. 2025;1760:466274. doi: 10.1016/j.chroma.2025.466274 40818431

[10] Müller E, Chung JT, Zhang Z, et al. Characterization of the mechanical properties of polymeric chromatographic particles by micromanipulation. J Chromatogr A. 2005;1097(1):116–123. doi: 10.1016/j.chroma.2005.08.014 16298190

[11] Claessens HA, van Straten MA. Review on the chemical and thermal stability of stationary phases for reversed-phase liquid chromatography. J Chromatogr A. 2004;1060(1):23–41. doi: 10.1016/j.chroma.2004.08.098 15628150

[12] Borges EM, Euerby MR. An appraisal of the chemical and thermal stability of silica based reversed-phase liquid chromatographic stationary phases employed within the pharmaceutical environment. J Pharm Biomed Anal. 2013;77:100–115. doi: 10.1016/j.jpba.2013.01.013 23411003

[13] Zhao J, Jiang ZT, Tan J, et al. Sol–gel synthesis and characterization of titania monolith with bimodal porosity. J Sol-Gel Sci Technol. 2011;58(2):436–441. doi: 10.1007/s10971-011-2410-2

[14] Nawrocki J, Dunlap C, McCormick A, et al. Part I. Chromatography using ultra-stable metal oxide-based stationary phases for HPLC. J Chromatogr A. 2004;1028(1):1–30. doi: 10.1016/j.chroma.2003.11.052 14969280

[15] Morteo-Flores F, Roldan A. The Effect of pristine and hydroxylated oxide surfaces on the guaiacol HDO process: a DFT study. Chemphyschem. 2022;23(1):e202100583. doi: 10.1002/cphc.202100583 34495572 PMC9292963

[16] Li J, Wang J, Yan Y, et al. Comprehensive evaluation of different TiO_2_-based phosphopeptide enrichment and fractionation Methods for phosphoproteomics. Cells. 2022;11(13):2047. doi: 10.3390/cells11132047 35805136 PMC9265536

[17] Ghose S, McNerney TM, Hubbard B. Preparative protein purification on underivatized silica. Biotechnol Bioeng. 2004;87(3):413–423. doi: 10.1002/bit.20125 15281115

[18] Schwaminger SP, Fraga-García P, Blank-Shim SA, et al. Magnetic One-step purification of His-tagged protein by Bare iron oxide nanoparticles. ACS Omega. 2019;4(2):3790–3799. doi: 10.1021/acsomega.8b03348 31459591 PMC6648446

[19] Hirano A, Kanoh S, Shiraki K, et al. Selective and high-capacity binding of immunoglobulin G to zirconia nanoparticles modified with phosphate groups. Colloids Surf B Biointerfaces. 2023;226:113291. doi: 10.1016/j.colsurfb.2023.113291 37031515

[20] Kanoh S, Tabata K, Saito S, et al. Purification of immunoglobulin a using mesoporous zirconia particles coated with phosphate. ACS Appl Mater Interface. 2025;17(36):50891–50900. doi: 10.1021/acsami.5c11319 40862504

[21] Kanoh S, Shiraki K, Wada M, et al. Chromatographic purification of histidine-tagged proteins using zirconia particles modified with phosphate groups. J Chromatogr A. 2023;1703:464112. doi: 10.1016/j.chroma.2023.464112 37285623

[22] Zhou H, Tian R, Ye M, et al. Highly specific enrichment of phosphopeptides by zirconium dioxide nanoparticles for phosphoproteome analysis. Electrophoresis. 2007;28(13):2201–2215. doi: 10.1002/elps.200600718 17539039

[23] Nelson CA, Szczech JR, Dooley CJ, et al. Effective enrichment and mass spectrometry analysis of phosphopeptides using mesoporous metal oxide nanomaterials. Anal Chem. 2010;82(17):7193–7201. doi: 10.1021/ac100877a 20704311 PMC2936271

[24] Nelson CA, Szczech JR, Xu Q, et al. Mesoporous zirconium oxide nanomaterials effectively enrich phosphopeptides for mass spectrometry-based phosphoproteomics. Chem Commun. 2009 43;(43):6607–6609. doi: 10.1039/B908788E PMC2790071 19865665

[25] Kweon HK, Håkansson K. Selective zirconium dioxide-based enrichment of phosphorylated peptides for mass spectrometric analysis. Anal Chem. 2006;78(6):1743–1749. doi: 10.1021/ac0522355 16536406

[26] Hertl W. Surface chemistry of zirconia polymorphs. Langmuir. 1989;5(1):96–100. doi: 10.1021/la00085a018

[27] Yang J, Youssef M, Yildiz B. Structure, kinetics, and thermodynamics of water and its ions at the Interface with monoclinic ZrO_2_ resolved via ab initio molecular dynamics. J Phys Chem C. 2021;125(28):15233–15242. doi: 10.1021/acs.jpcc.1c02064

[28] Sugiyama N, Masuda T, Shinoda K, et al. Phosphopeptide enrichment by aliphatic hydroxy acid-modified metal oxide chromatography for nano-LC-MS/MS in Proteomics applications*S. Mol Cellular Proteomics. 2007;6(6):1103–1109. doi: 10.1074/mcp.T600060-MCP200 17322306

[29] Li XS, Chen X, Yuan BF, et al. Phosphonate-modified metal oxides for the highly selective enrichment of phosphopeptides. RSC Adv. 2015;5(11):7832–7841. doi: 10.1039/C4RA13878C

[30] Okuda T, Kitamura M, Kato K. A zirconia-based column chromatography system optimized for the purification of IgM from hybridoma culture supernatants. Anal Biochem. 2022;657:114900. doi: 10.1016/j.ab.2022.114900 36122604

[31] Okuda T, Kato K, Kitamura M, et al. Purification of anti-glycoconjugate monoclonal antibodies using newly developed porous zirconia particles. Sci Rep. 2021;11(1):3233. doi: 10.1038/s41598-021-82457-0 33564002 PMC7873262

[32] Subramanian A, Carr PW, McNeff CV. Use of spray-dried zirconia microspheres in the separation of immunoglobulins from cell culture supernatant. J Chromatogr A. 2000;890(1):15–23. doi: 10.1016/S0021-9673(00)00289-2 10976790

[33] Schafer WA, Carr PW. Chromatographic characterization of a phosphate-modified zirconia support for bio-chromatographic applications. J Chromatogr A. 1991;587(2):149–160. doi: 10.1016/0021-9673(91)85151-5 1783667

[34] Okuda T, Kato K. A simple procedure for preparing biotinylated immunoglobulin M from hybridoma culture medium. Protein Expr Purif. 2025;225:106595. doi: 10.1016/j.pep.2024.106595 39197671

[35] Liu H, Zhou J, Huang H. Amine-functionalized TiO_2_ nanoparticles for highly selective enrichment of phosphopeptides. Talanta. 2015;143:431–437. doi: 10.1016/j.talanta.2015.05.019 26078180

[36] Lu Z, Duan J, He L, et al. Mesoporous TiO_2_ nanocrystal clusters for selective enrichment of phosphopeptides. Anal Chem. 2010;82(17):7249–7258. doi: 10.1021/ac1011206 20712324

[37] Dong Y, Laaksonen A, Gong M, et al. Selective separation of highly similar proteins on ionic liquid-loaded mesoporous TiO_2_. Langmuir. 2022;38(10):3202–3211. doi: 10.1021/acs.langmuir.1c03277 35253426 PMC8928471

[38] Tang J, Yin P, Lu X, et al. Development of mesoporous TiO_2_ microspheres with high specific surface area for selective enrichment of phosphopeptides by mass spectrometric analysis. J Chromatogr A. 2010;1217(15):2197–2205. doi: 10.1016/j.chroma.2010.02.008 20219200

[39] Martra G. Lewis acid and base sites at the surface of microcrystalline TiO_2_ anatase: relationships between surface morphology and chemical behaviour. Appl Catal Gen. 2000;200(1):275–285. doi: 10.1016/S0926-860X(00)00641-4

[40] Hosseinpour S, Tang F, Wang F, et al. Chemisorbed and physisorbed water at the TiO_2_/water interface. J Phys Chem Lett. 2017;8(10):2195–2199. doi: 10.1021/acs.jpclett.7b00564 28447795 PMC5489252

[41] Kavathekar RS, English NJ, MacElroy JMD, MacElroy JMD. Spatial distribution of adsorbed water layers at the TiO_2_ rutile and anatase interfaces. Chem Phys Lett. 2012;554:102–106. doi: 10.1016/j.cplett.2012.10.004

[42] Tape CJ, Worboys JD, Sinclair J, et al. Reproducible automated phosphopeptide enrichment using magnetic TiO_2_ and Ti-IMAC. Anal Chem. 2014;86(20):10296–10302. doi: 10.1021/ac5025842 25233145 PMC4206527

[43] Pocsfalvi G, Cuccurullo M, Schlosser G, et al. Phosphorylation of B14.5a subunit from bovine heart complex I identified by titanium dioxide selective enrichment and shotgun Proteomics*S. Mol Cellular Proteomics. 2007;6(2):231–237. doi: 10.1074/mcp.M600268-MCP200 17114648

[44] Larsen MR, Thingholm TE, Jensen ON, et al. Highly selective enrichment of phosphorylated peptides from peptide mixtures using titanium dioxide microcolumns*. Mol Cellular Proteomics. 2005;4(7):873–886. doi: 10.1074/mcp.T500007-MCP200 15858219

[45] Fukuda I, Hirabayashi-Ishioka Y, Sakikawa I, et al. Optimization of enrichment conditions on TiO_2_ chromatography using glycerol as an additive reagent for effective phosphoproteomic analysis. J Proteome Res. 2013;12(12):5587–5597. doi: 10.1021/pr400546u 24245541

[46] Moerz ST, Huber P. pH-dependent selective protein adsorption into mesoporous silica. J Phys Chem C. 2015;119(48):27072–27079. doi: 10.1021/acs.jpcc.5b09606

[47] Soto-Rodríguez J, Coyle BL, Samuelson A, et al. Affinity purification of Car9-tagged proteins on silica matrices: optimization of a rapid and inexpensive protein purification technology. Protein Expr Purif. 2017;135:70–77. doi: 10.1016/j.pep.2017.05.003 28506644

[48] Wu JH, Li XS, Zhao Y, et al. Application of liquid phase deposited titania nanoparticles on silica spheres to phosphopeptide enrichment and high performance liquid chromatography packings. J Chromatogr A. 2011;1218(20):2944–2953. doi: 10.1016/j.chroma.2011.03.019 21470615

[49] Gao M, Liu Q, Xue Y, et al. Facile synthesis of peanut-like Sn-doped silica nano-adsorbent for affinity separation of proteins. RSC Adv. 2022;12(8):4697–4702. doi: 10.1039/D1RA08362G 35425506 PMC8981230

[50] Ikeda T, Motomura K, Agou Y, et al. The silica-binding Si-tag functions as an affinity tag even under denaturing conditions. Protein Expr Purif. 2011;77(2):173–177. doi: 10.1016/j.pep.2011.01.012 21277372

[51] Ikeda T, Ninomiya KI, Hirota R, et al. Single-step affinity purification of recombinant proteins using the silica-binding Si-tag as a fusion partner. Protein Expr Purif. 2010;71(1):91–95. doi: 10.1016/j.pep.2009.12.009 20034569

[52] Abdelhamid MAA, Motomura K, Ikeda T, et al. Affinity purification of recombinant proteins using a novel silica-binding peptide as a fusion tag. Appl Microbiol Biotechnol. 2014;98(12):5677–5684. doi: 10.1007/s00253-014-5754-z 24756322

[53] Freitas AI, Domingues L, Aguiar TQ. Bare silica as an alternative matrix for affinity purification/immobilization of His-tagged proteins. Sep Purif Technol. 2022;286:120448. doi: 10.1016/j.seppur.2022.120448

[54] Lowe CR, Glad M, Larsson PO, et al. High-performance liquid affinity chromatography of proteins on Cibacron Blue F3G-A bonded silica. J Chromatogr A. 1981;215:303–316. doi: 10.1016/S0021-9673(00)81409-0

[55] Yang Y, Qu Q, Li W, et al. Preparation of a silica-based high-performance hydrophobic interaction chromatography stationary phase for protein separation and renaturation. J Sep Sci. 2016;39(13):2481–2490. doi: 10.1002/jssc.201501216 27159821

[56] Tian Y, Tang R, Liu L, et al. Glutathione-modified ordered mesoporous silicas for enrichment of N-linked glycopeptides by hydrophilic interaction chromatography. Talanta. 2020;217:121082. doi: 10.1016/j.talanta.2020.121082 32498907

[57] Salimi K, Usta DD, Koçer İ, et al. Protein a and protein A/G coupled magnetic SiO_2_ microspheres for affinity purification of immunoglobulin G. Int J Biol Macromol. 2018;111:178–185. doi: 10.1016/j.ijbiomac.2018.01.019 29309863

[58] Wan H, Yan J, Yu L, et al. Zirconia layer coated mesoporous silica microspheres used for highly specific phosphopeptide enrichment. Talanta. 2010;82(5):1701–1707. doi: 10.1016/j.talanta.2010.07.050 20875566

[59] Liao Y, Cheng Y, Li Q. Preparation of nitrilotriacetic acid/Co^2+^-linked, silica/boron-coated magnetite nanoparticles for purification of 6×histidine-tagged proteins. J Chromatogr A. 2007;1143(1):65–71. doi: 10.1016/j.chroma.2006.12.063 17204270

[60] Lion M, Maache M, Lavalley JC, et al. FT-IR study of the brønsted acidity of phosphated and sulphated silica catalysts. J Mol Struct. 1990;218:417–422. doi: 10.1016/0022-2860(90)80303-2

[61] Schrader AM, Monroe JI, Sheil R, et al. Surface chemical heterogeneity modulates silica surface hydration. Proc Natl Acad Sci. 2018;115(12):2890–2895. doi: 10.1073/pnas.1722263115 29507240 PMC5866604

[62] Gierada M, De Proft F, Sulpizi M, et al. Understanding the acidic properties of the amorphous hydroxylated silica surface. J Phys Chem C. 2019;123(28):17343–17352. doi: 10.1021/acs.jpcc.9b04137

[63] Mehyou Z, Lobinski R, Hagège A. One-step coating of silica capillaries for selective protein retention by Cu(II)-IDA IMAC. Talanta. 2011;87:168–173. doi: 10.1016/j.talanta.2011.09.057 22099664

[64] Miller NT, Shieh CH. High-performance anion-exchange chromatography of proteins using aza-ether bonded silica-based phases. J Chromatogr A. 1989;463:329–344. doi: 10.1016/S0021-9673(01)84487-3 2708487

[65] Lei G, Xiong X, Wei Y, et al. Novel tetrazole-functionalized ion exchanger for weak cation-exchange chromatography of proteins. J Chromatogr A. 2008;1187(1):197–204. doi: 10.1016/j.chroma.2008.02.031 18313064

[66] Itoh H, Nimura N, Kinoshita T, et al. Fast protein separation by reversed-phase high-performance liquid chromatography on octadecylsilyl-bonded nonporous silica gel: II. Improvement in recovery of hydrophobic proteins. Anal Biochem. 1991;199(1):7–10. doi: 10.1016/0003-2697(91)90261-Q 1666942

[67] Xu Z, Uddin KMA, Kamra T, et al. Fluorescent boronic acid polymer grafted on silica particles for affinity separation of Saccharides. ACS Appl Mater Interface. 2014;6(3):1406–1414. doi: 10.1021/am405531n PMC3963438 24444898

[68] Gu F, Chodavarapu K, McCreary D, et al. Silica-based strong anion exchange media for protein purification. J Chromatogr A. 2015;1376:53–63. doi: 10.1016/j.chroma.2014.11.082 25537176

[69] Gupta S, Pfannkoch E, Regnier FE. High-performance cation-exchange chromatography of proteins. Anal Biochem. 1983;128(1):196–201. doi: 10.1016/0003-2697(83)90363-9 6303152

[70] Huang S, Cheng SY, Zhang SY, et al. Protein A-mesoporous silica composites for chromatographic purification of immunoglobulin G. New J Chem. 2020;44(19):7884–7890. doi: 10.1039/D0NJ00337A

[71] Liu Y, Fu D, Xiao Y, et al. Synthesis and evaluation of a silica-bonded concanavalin a material for lectin affinity enrichment of N-linked glycoproteins and glycopeptides. Analytical Methods. 2015;7(1):25–28. doi: 10.1039/C4AY02286F

[72] Naik AD, Islam T, Terasaka T, et al. Silica resins and peptide ligands to develop disposable affinity adsorbents for antibody purification. Biochem Eng J. 2019;145:53–61. doi: 10.1016/j.bej.2018.07.011

[73] Furuya M, Tsushima Y, Tani S, et al. Development of affinity chromatography using a bioactive peptide as a ligand. Bioorg Med Chem. 2006;14(15):5093–5098. doi: 10.1016/j.bmc.2006.04.020 16650997

[74] Rauwolf S, Steegmüller T, Schwaminger SP, et al. Purification of a peptide tagged protein via an affinity chromatographic process with underivatized silica. Eng Life Sci. 2021;21(10):549–557. doi: 10.1002/elsc.202100019 34690628 PMC8518568

[75] Coyle BL, Baneyx F. A cleavable silica-binding affinity tag for rapid and inexpensive protein purification. Biotechnol Bioeng. 2014;111(10):2019–2026. doi: 10.1002/bit.25257 24777569

[76] Lee A, Yang HJ, Lim ES, et al. Enrichment of phosphopeptides using bare magnetic particles. Rapid Commun Mass Spectrom. 2008;22(16):2561–2564. doi: 10.1002/rcm.3652 18655002

[77] Aguiar TQ, Domingues L. Recombinant protein purification and immobilization strategies based on peptides with dual affinity to iron oxide and silica. Biotechnol J. 2023;18(11):2300152. doi: 10.1002/biot.202300152 37478356

[78] Yang J, Ni K, Wei D, et al. One-step purification and immobilization of his-tagged protein via Ni^2+^-functionalized Fe_3_O_4_@polydopamine magnetic nanoparticles. Biotechnol Bioprocess Eng. 2015;20(5):901–907. doi: 10.1007/s12257-015-0136-7

[79] Zhang X, Wang J, He X, Chen L, Zhang Y. Tailor-made boronic acid functionalized magnetic nanoparticles with a tunable polymer shell-assisted for the selective enrichment of glycoproteins/glycopeptides. ACS Appl Mater Interface. 2015;7(44):24576–24584. doi: 10.1021/acsami.5b06445 26479332

[80] Kim S, Sung D, Chang JH. Highly efficient antibody purification with controlled orientation of protein a on magnetic nanoparticles. Medchemcomm. 2018;9(1):108–112. doi: 10.1039/C7MD00468K 30108904 PMC6072530

[81] Zhang J, He T, Tang L, et al. Boronic acid functionalized Fe_3_O_4_ magnetic microspheres for the specific enrichment of glycoproteins. J Sep Sci. 2016;39(9):1691–1699. doi: 10.1002/jssc.201500921 27171767

[82] Wang Y, Wei Y, Gao P, et al. Preparation of Fe_3_O_4_@PMAA@Ni microspheres towards the efficient and selective enrichment of histidine-rich proteins. ACS Appl Mater Interface. 2021;13(9):11166–11176. doi: 10.1021/acsami.0c19734 33635047

[83] Li Y, Leng T, Lin H, et al. Preparation of Fe_3_O_4_@ZrO_2_ core−Shell microspheres as affinity probes for selective enrichment and direct determination of phosphopeptides using matrix-assisted laser desorption ionization mass spectrometry. J Proteome Res. 2007;6(11):4498–4510. doi: 10.1021/pr070167s 17900103

[84] Chen CT, Chen YC. Fe_3_O_4_/TiO_2_ core/shell nanoparticles as affinity probes for the analysis of phosphopeptides using TiO_2_ surface-assisted Laser desorption/Ionization mass spectrometry. Anal Chem. 2005;77(18):5912–5919. doi: 10.1021/ac050831t 16159121

[85] Ni Q, Chen B, Dong S, et al. Preparation of core–shell structure Fe_3_O_4_@SiO_2_ superparamagnetic microspheres immoblized with iminodiacetic acid as immobilized metal ion affinity adsorbents for His-tag protein purification. Biomed Chromatogr. 2016;30(4):566–573. doi: 10.1002/bmc.3584 26268650

[86] Mulakaluri N, Pentcheva R, Scheffler M. Coverage-dependent adsorption mode of water on Fe3O4(001): insights from first principles calculations. J Phys Chem C. 2010;114(25):11148–11156. doi: 10.1021/jp100344n

[87] Jolsterå R, Gunneriusson L, Holmgren A. Surface complexation modeling of Fe_3_O_4_–H^+^ and Mg(II) sorption onto maghemite and magnetite. J Colloid Interface Sci. 2012;386(1):260–267. doi: 10.1016/j.jcis.2012.07.031 22889624

[88] Jose L, Lee C, Hwang A, et al. Magnetically steerable Fe_3_O_4_@Ni^2+^-NTA-polystyrene nanoparticles for the immobilization and separation of his6-protein. Eur Polym J. 2019;112:524–529. doi: 10.1016/j.eurpolymj.2019.01.024

[89] Slováková M, Sedlák M, Křížková B, et al. Application of trypsin Fe_3_O_4_@SiO_2_ core/shell nanoparticles for protein digestion. Process Biochem. 2015;50(12):2088–2098. doi: 10.1016/j.procbio.2015.09.002

[90] Laurent CJCM, Billiet HAH, De Galan L, et al. High-performance liquid chromatography of proteins on alumina. J Chromatogr A. 1984;287:45–54. doi: 10.1016/S0021-9673(01)87672-X

[91] Li Y, Liu Y, Tang J, et al. Fe_3_O_4_@Al_2_O_3_ magnetic core–shell microspheres for rapid and highly specific capture of phosphopeptides with mass spectrometry analysis. J Chromatogr A. 2007;1172(1):57–71. doi: 10.1016/j.chroma.2007.09.062 17936290

[92] Haneda M, Joubert E, Ménézo JC, et al. Surface characterization of alumina-supported catalysts prepared by sol–gel method. Part I.—acid–base properties. Phys Chem Chem Phys. 2001;3(7):1366–1370. doi: 10.1039/B009945G

[93] Yang X, Sun Z, Wang D, et al. Surface acid–base properties and hydration/dehydration mechanisms of aluminum (hydr)oxides. J Colloid Interface Sci. 2007;308(2):395–404. doi: 10.1016/j.jcis.2006.12.023 17275018

[94] Haky JE, Raghani A, Dunn BM. Comparison of polybutadiene-coated alumina and octadecyl-bonded silica for separations of proteins and peptides by reversed-phase high-performance liquid chromatography. J Chromatogr A. 1991;541:303–315. doi: 10.1016/S0021-9673(01)96002-9

[95] Haky JE, Raghani AR, Dunn BM, et al. Evaluation of octadecyl-bonded alumina for separations of proteins and peptides by reversed-phase high-performance liquid chromatography. Chromatographia. 1991;32(1):49–55. doi: 10.1007/BF02262466

[96] Bhogale A, Patel N, Sarpotdar P, et al. Systematic investigation on the interaction of bovine serum albumin with ZnO nanoparticles using fluorescence spectroscopy. Colloids Surf B Biointerfaces. 2013;102:257–264. doi: 10.1016/j.colsurfb.2012.08.023 23010116

[97] Bukackova M, Marsalek R. Interaction of BSA with ZnO, TiO_2_, and CeO_2_ nanoparticles. Biophys Chem. 2020;267:106475. doi: 10.1016/j.bpc.2020.106475 32950875

[98] Cheng YH, Lai CM, Lin KS, et al. Effects of metal oxide nanoparticles on the structure and activity of lysozyme. Colloids Surf B Biointerfaces. 2017;151:344–353. doi: 10.1016/j.colsurfb.2016.12.030 28043051

[99] Šimšíková M, Antalík M. Interaction of cytochrome c with zinc oxide nanoparticles. Colloids Surf B Biointerfaces. 2013;103:630–634. doi: 10.1016/j.colsurfb.2012.10.058 23274157

[100] Sasidharan NP, Chandran P, Sudheer Khan S. Interaction of colloidal zinc oxide nanoparticles with bovine serum albumin and its adsorption isotherms and kinetics. Colloids Surf B Biointerfaces. 2013;102:195–201. doi: 10.1016/j.colsurfb.2012.07.034 23000680

[101] Mahajan M, Kumar S, Gaur J, et al. Green synthesis of ZnO nanoparticles using justicia adhatoda for photocatalytic degradation of malachite green and reduction of 4-nitrophenol. RSC Adv. 2025;15(4):2958–2980. doi: 10.1039/D4RA08632E 39881999 PMC11775505

[102] Yıldırım D, Gökçal B, Büber E, et al. A new nanozyme with peroxidase-like activity for simultaneous phosphoprotein isolation and detection based on metal oxide affinity chromatography: monodisperse-porous cerium oxide microspheres. Chem Eng J. 2021;403:126357. doi: 10.1016/j.cej.2020.126357

[103] Sun S, Ma H, Han G, et al. Efficient enrichment and identification of phosphopeptides by cerium oxide using on-plate matrix-assisted laser desorption/ionization time-of-flight mass spectrometric analysis. Rapid Commun Mass Spectrom. 2011;25(13):1862–1868. doi: 10.1002/rcm.5055 21638362

[104] Wu Z, Mann AKP, Li M, et al. Spectroscopic investigation of surface-dependent acid–base property of Ceria Nanoshapes. J Phys Chem C. 2015;119(13):7340–7350. doi: 10.1021/acs.jpcc.5b00859

[105] Zhang G, Zhou Y, Yang Y, et al. Elucidating the role of surface Ce^4+^ and oxygen vacancies of CeO_2_ in the direct synthesis of dimethyl carbonate from CO_2_ and methanol. Molecules. 2023;28(9):3785. doi: 10.3390/molecules28093785 37175195 PMC10180377

[106] Sun DH, Zhang JL. Simulated calculation of surface acid-base equilibrium constants and surface species distribution of the nanoceria suspension. Appl Surf Sci. 2019;495:143563. doi: 10.1016/j.apsusc.2019.143563

[107] Sturm M, Leitner A, Smått JH, et al. Tin dioxide microspheres as a promising material for phosphopeptide enrichment prior to liquid chromatography-(tandem) mass spectrometry analysis. Adv Funct Mater. 2008;18(16):2381–2389. doi: 10.1002/adfm.200800215

[108] Leitner A, Sturm M, Smått JH, et al. Optimizing the performance of tin dioxide microspheres for phosphopeptide enrichment. Anal Chim Acta. 2009;638(1):51–57. doi: 10.1016/j.aca.2009.01.063 19298879

[109] Zhang W, Lin Z, Li H, et al. Surface acidity of tin dioxide nanomaterials revealed with ^31^P solid-state NMR spectroscopy and DFT calculations. RSC Adv. 2021;11(40):25004–25009. doi: 10.1039/D1RA02782D 35481043 PMC9037001

[110] Ficarro SB, Parikh JR, Blank NC, et al. Niobium(V) oxide (Nb_2_O_5_): application to phosphoproteomics. Anal Chem. 2008;80(12):4606–4613. doi: 10.1021/ac800564h 18491922

[111] Nakajima K, Baba Y, Noma R, et al. Nb_2_O_5_·nH_2_O as a heterogeneous catalyst with water-tolerant Lewis acid sites. J Am Chem Soc. 2011;133(12):4224–4227. doi: 10.1021/ja110482r 21370861

[112] Qi D, Lu J, Deng C, et al. Development of core–shell structure Fe_3_O_4_@Ta_2_O_5_ microspheres for selective enrichment of phosphopeptides for mass spectrometry analysis. J Chromatogr A. 2009;1216(29):5533–5539. doi: 10.1016/j.chroma.2009.05.049 19515374

[113] Kondo JN, Lu L, Takahara Y, et al. IR characterization of mesoporous tantalum oxide, Ta-TMS-1. Bull Chem Soc Jpn. 2000;73(5):1123–1129. doi: 10.1246/bcsj.73.1123

[114] Li Y, Lin H, Deng C, et al. Highly selective and rapid enrichment of phosphorylated peptides using gallium oxide-coated magnetic microspheres for MALDI-TOF-MS and nano-LC-ESI-MS/MS/MS analysis. Proteomics. 2008;8(2):238–249. doi: 10.1002/pmic.200700454 18081192

[115] Castro-Fernández P, Mance D, Liu C, et al. Propane dehydrogenation on Ga_2_O_3_-based catalysts: contrasting performance with coordination environment and acidity of surface sites. ACS Catal. 2021;11(2):907–924. doi: 10.1021/acscatal.0c05009

[116] Rodríguez Delgado M, Otero Areán C. Surface chemistry and pore structure of β-Ga_2_O_3_. Mater Lett. 2003;57(15):2292–2297. doi: 10.1016/S0167-577X(02)01214-4

[117] Wu Y, Gao F, Wang H, et al. Probing acid–base properties of anatase TiO_2_ nanoparticles with dominant {001} and {101} facets using methanol chemisorption and surface reactions. J Phys Chem C. 2021;125(7):3988–4000. doi: 10.1021/acs.jpcc.0c11107

[118] Xu F, Chen W, Walenta CA, et al. Dual Lewis site creation for activation of methanol on Fe_3_O_4_(111) thin films. Chem Sci. 2020;11(9):2448–2454. doi: 10.1039/C9SC06149E 34084409 PMC8157392

[119] Wöll C. The chemistry and physics of zinc oxide surfaces. Prog Surf Sci. 2007;82(2):55–120. doi: 10.1016/j.progsurf.2006.12.002

[120] Vimont A, Lavalley JC, Sahibed-Dine A, et al. Infrared spectroscopic study on the surface properties of γ-gallium oxide as compared to those of γ-alumina. J Phys Chem B. 2005;109(19):9656–9664. doi: 10.1021/jp050103+ 16852163

[121] Kosmulski M. The pH-dependent surface charging and points of zero charge: V. J Colloid Interface Sci. 2011;353(1):1–15. doi: 10.1016/j.jcis.2010.08.023 20869721

[122] Sulpizi M, Gaigeot MP, Sprik M. The silica–water Interface: how the silanols determine the surface acidity and modulate the water properties. J Chem Theory Comput. 2012;8(3):1037–1047. doi: 10.1021/ct2007154 26593364

[123] Gaigeot MP, Sprik M, Sulpizi M. Oxide/Water interfaces: how the surface chemistry modifies interfacial water properties. J Phys: Condens Matter. 2012;24(12):124106. doi: 10.1088/0953-8984/24/12/124106 22395098

[124] Bañuelos JL, Borguet E, Brown GE Jr, et al. Oxide– and silicate–water interfaces and their roles in Technology and the environment. Chem Rev. 2023;123(10):6413–6544. doi: 10.1021/acs.chemrev.2c00130 37186959

[125] Nagai S, Urata S, Suga K, et al. Three-dimensional ordering of water molecules reflecting hydroxyl groups on sapphire (001) and α-quartz (100) surfaces. Nanoscale. 2023;15(32):13262–13271. doi: 10.1039/D3NR02498A 37539559

[126] Skelton AA, Wesolowski DJ, Cummings PT. Investigating the quartz (101̅0)/Water Interface using classical and ab initio molecular dynamics. Langmuir. 2011;27(14):8700–8709. doi: 10.1021/la2005826 21648451

[127] Wu Q, Chen J, Ren Y, et al. Species identification of phosphate at the ZrO_2_/water interface: a combined ATR-FTIR and DFT study. Appl Surf Sci. 2022;606:154946. doi: 10.1016/j.apsusc.2022.154946

[128] Bérard R, Sassoye C, Terrisse H, et al. Effect of crystalline phase and facet nature on the adsorption of phosphate species onto TiO_2_ nanoparticles. Langmuir. 2024;40(31):16258–16271. doi: 10.1021/acs.langmuir.4c01447 39039729

[129] Lehman SE, Mudunkotuwa IA, Grassian VH, et al. Nano–bio interactions of porous and nonporous silica nanoparticles of varied surface chemistry: a structural, kinetic, and thermodynamic study of protein adsorption from RPMI culture medium. Langmuir. 2016;32(3):731–742. doi: 10.1021/acs.langmuir.5b03997 26716353

[130] Katiyar A, Ji L, Smirniotis P, et al. Protein adsorption on the mesoporous molecular sieve silicate SBA-15: effects of pH and pore size. J Chromatogr A. 2005;1069(1):119–126. doi: 10.1016/j.chroma.2004.10.077 15844490

[131] Clemments AM, Botella P, Landry CC. Protein adsorption from biofluids on silica nanoparticles: corona analysis as a function of particle diameter and porosity. ACS Appl Mater Interface. 2015;7(39):21682–21689. doi: 10.1021/acsami.5b07631 PMC5084906 26371804

[132] Wardani RK, Dahlan K, Wahyudi ST, et al. Synthesis and characterization of nanoparticle magnetite for biomedical application. AIP Conf Proc. 2019;2194(1):020137. doi: 10.1063/1.5139869

[133] Ouyang F, Nakayama A, Tabada K, et al. Infrared study of a novel acid−Base site on ZrO_2_ by adsorbed probe molecules. I. Pyridine, carbon dioxide, and formic acid adsorption. J Phys Chem B. 2000;104(9):2012–2018. doi: 10.1021/jp992970i

[134] Tanabe K, Yamaguchi T. Acid-base bifunctional catalysis by ZrO_2_ and its mixed oxides. Catal Today. 1994;20(2):185–197. doi: 10.1016/0920-5861(94)80002-2

[135] Yashima M, Mitsuhashi T, Takashina H, et al. Tetragonal—monoclinic phase transition enthalpy and temperature of ZrO_2_-CeO_2_ solid solutions. J Am Ceramic Soc. 1995;78(8):2225–2228. doi: 10.1111/j.1151-2916.1995.tb08642.x

[136] Pokrovski K, Jung KT, Bell AT. Investigation of CO and CO_2_ adsorption on tetragonal and monoclinic zirconia. Langmuir. 2001;17(14):4297–4303. doi: 10.1021/la001723z

[137] Tanabe K. Surface and catalytic properties of ZrO_2_. Mater Chem Phys. 1985;13(3):347–364. doi: 10.1016/0254-0584(85)90064-1

[138] Hirano A, Wada M, Kitamura M, et al. Interactions between amino acids and zirconia modified with Ethylenediaminetetra(methylenephosphonic acid): mechanistic insights into the selective binding of antibodies. Langmuir. 2021;37(4):1605–1612. doi: 10.1021/acs.langmuir.0c03471 33478221

[139] Zhao S, Hu W, Zhang D, et al. Packed in-tube solid-phase microextraction with mesoporous zirconium titanium oxides for highly efficient phosphopeptide enrichment and analysis. Microchem J. 2023;195:109423. doi: 10.1016/j.microc.2023.109423

[140] Block H, Maertens B, Spriestersbach A, et al. Chapter 27 immobilized-metal affinity chromatography (IMAC): a review. In: Burgess RR, Deutscher MP, editors. Methods in Enzymology. Vol. 463. San Diego (CA): Academic Press; 2009. p. 439–473. doi: 10.1016/S0076-6879(09)63027-5 19892187

[141] Qu J, Li Y, Wan Y. Recommendation of a milder cleaning-in-place method for Cytiva’s second-generation protein L resin MabSelect VL. J Chromatogr B. 2025;1267:124810. doi: 10.1016/j.jchromb.2025.124810 41061491

[142] Yang L, Harding JD, Ivanov AV, et al. Effect of cleaning agents and additives on protein a ligand degradation and chromatography performance. J Chromatogr A. 2015;1385:63–68. doi: 10.1016/j.chroma.2015.01.068 25680549

[143] Maleki F, Di Liberto G, Pacchioni G. pH- and facet-dependent surface chemistry of TiO_2_ in aqueous environment from First principles. ACS Appl Mater Interface. 2023;15(8):11216–11224. doi: 10.1021/acsami.2c19273 PMC9982820 36786774

[144] Paunović V, Rellán-Piñeiro M, López N, et al. Activity differences of rutile and anatase TiO_2_ polymorphs in catalytic HBr oxidation. Catal Today. 2021;369:221–226. doi: 10.1016/j.cattod.2020.03.036

[145] Liu G, Yan X, Chen Z, et al. Synthesis of rutile–anatase core–shell structured TiO_2_ for photocatalysis. J Mater Chem. 2009;19(36):6590–6596. doi: 10.1039/B902666E

[146] Huangfu C, Dong Y, Ji X, et al. Mechanistic study of protein adsorption on mesoporous TiO_2_ in aqueous Buffer solutions. Langmuir. 2019;35(34):11037–11047. doi: 10.1021/acs.langmuir.9b01354 31378070

[147] Wang B, Xie Z, Ding CF, et al. Recent advances in metal oxide affinity chromatography materials for phosphoproteomics. Trends Analyt Chem. 2023;158:116881. doi: 10.1016/j.trac.2022.116881

[148] Weisz AD, Regazzoni AE, Blesa MA. ATR–FTIR study of the stability trends of carboxylate complexes formed on the surface of titanium dioxide particles immersed in water. Solid State Ion. 2001;143(1):125–130. doi: 10.1016/S0167-2738(01)00840-2

[149] Tielens F, Gervais C, Deroy G, et al. Characterization of phosphate species on hydrated anatase TiO_2_ surfaces. Langmuir. 2016;32(4):997–1008. doi: 10.1021/acs.langmuir.5b03519 26734828

[150] Agosta L, Zollo G, Arcangeli C, et al. Water driven adsorption of amino acids on the (101) anatase TiO_2_ surface: an ab initio study. Phys Chem Chem Phys. 2015;17(3):1556–1561. doi: 10.1039/C4CP03056G 25434879

[151] Liu S, Meng XY, Perez-Aguilar JM, et al. An in silico study of TiO_2_ nanoparticles interaction with twenty standard amino acids in aqueous solution. Sci Rep. 2016;6(1):37761. doi: 10.1038/srep37761 27883086 PMC5121885

[152] Thingholm TE, Jørgensen TJD, Jensen ON, et al. Highly selective enrichment of phosphorylated peptides using titanium dioxide. Nat Protoc. 2006;1(4):1929–1935. doi: 10.1038/nprot.2006.185 17487178

[153] Fíla J, Honys D. Enrichment techniques employed in phosphoproteomics. Amino Acids. 2012;43(3):1025–1047. doi: 10.1007/s00726-011-1111-z 22002794 PMC3418503

[154] Pocsfalvi G. Chapter 5 selective enrichment in phosphopeptides for the identification of phosphorylated mitochondrial proteins. In: Allison WS, Murphy A, editors. Methods in enzymology. Vol. 457. San Diego (CA): Academic Press; 2009. p. 81–96. doi: 10.1016/S0076-6879(09)05005-8 19426863

[155] Vansant EF, Van Der Voort P, Vrancken KC, editors. Chapter 3 the surface chemistry of silica. In: Studies in surface science and catalysis. Vol 93. Amsterdam: Elsevier; 1995. p. 59–77. doi: 10.1016/S0167-2991(06)81511-9

[156] Rimola A, Costa D, Sodupe M, et al. Silica surface features and their role in the adsorption of biomolecules: computational modeling and experiments. Chem Rev. 2013;113(6):4216–4313. doi: 10.1021/cr3003054 23289428

[157] Bousse L, Mostarshed S, Van Der Shoot B, et al. Zeta potential measurements of Ta_2_O_5_ and SiO_2_ thin films. J Colloid Interface Sci. 1991;147(1):22–32. doi: 10.1016/0021-9797(91)90130-Z

[158] Graffius G, Bernardoni F, Fadeev AY. Covalent functionalization of silica surface using “inert. Poly(dimethylsiloxanes). Langmuir. 2014;30(49):14797–14807. doi: 10.1021/la5031763 25419641

[159] Gondim DR, Cecilia JA, Rodrigues TNB, et al. Protein adsorption onto modified porous silica by single and binary human serum protein solutions. Int J Mol Sci. 2021;22(17):9164. doi: 10.3390/ijms22179164 34502072 PMC8430731

[160] Nawrocki J. The silanol group and its role in liquid chromatography. J Chromatogr A. 1997;779(1):29–71. doi: 10.1016/S0021-9673(97)00479-2

[161] Kitadai N, Yokoyama T, Nakashima S. ATR-IR spectroscopic study of L-lysine adsorption on amorphous silica. J Colloid Interface Sci. 2009;329(1):31–37. doi: 10.1016/j.jcis.2008.09.072 18930240

[162] McUmber AC, Randolph TW, Schwartz DK. Electrostatic interactions influence protein adsorption (but not desorption) at the silica–aqueous Interface. J Phys Chem Lett. 2015;6(13):2583–2587. doi: 10.1021/acs.jpclett.5b00933 26266737

[163] Goumans TPM, Wander A, Brown WA, et al. Structure and stability of the (001) α-quartz surface. Phys Chem Chem Phys. 2007;9(17):2146–2152. doi: 10.1039/b701176h 17464397

[164] Moitra N, Ichii S, Kamei T, et al. Surface functionalization of silica by Si–H activation of Hydrosilanes. J Am Chem Soc. 2014;136(33):11570–11573. doi: 10.1021/ja504115d 25101719

[165] Huang DM, Cao DB, Li YW, et al. Density function theory study of CO adsorption on Fe_3_O_4_(111) surface. J Phys Chem B. 2006;110(28):13920–13925. doi: 10.1021/jp0568273 16836342

[166] Hou S, Fang D, Jin Q, et al. First principle study on the interactions of NH_3_, NO_x_, and O_2_ with Fe_3_O_4_ (111) surfaces. Appl Surf Sci. 2022;605:154645. doi: 10.1016/j.apsusc.2022.154645

[167] Persson P, Nilsson N, Sjöberg S. Structure and bonding of orthophosphate ions at the iron oxide–aqueous Interface. J Colloid Interface Sci. 1996;177(1):263–275. doi: 10.1006/jcis.1996.0030 10479441

[168] Gamba O, Noei H, Pavelec J, et al. Adsorption of formic acid on the Fe_3_O_4_(001) surface. J Phys Chem C. 2015;119(35):20459–20465. doi: 10.1021/acs.jpcc.5b05560

[169] Klotz S, Steinle-Neumann G, Strässle TH, et al. Magnetism and the Verwey transition in Fe_3_O_4_ under pressure. Phys Rev B. 2008;77(1):12411. doi: 10.1103/PhysRevB.77.012411

[170] Nguyen MD, Tran HV, Xu S, et al. Fe_3_O_4_ nanoparticles: structures, synthesis, magnetic properties, surface functionalization, and emerging applications. Appl Sci. 2021;11(23):11301. doi: 10.3390/app112311301 35844268 PMC9285867

[171] Porath J. Immobilized metal ion affinity chromatography. Protein Expr Purif. 1992;3(4):263–281. doi: 10.1016/1046-5928(92)90001-D 1422221

[172] Wang G, Pan Z, Zhu X, et al. Mesoporous magnetic nanoparticles conjugated aptamers for exosomes capture and detection of Alzheimer’s disease. Engineered Regener. 2023;4(4):349–356. doi: 10.1016/j.engreg.2023.04.007

[173] Busca G. Chapter three - structural, surface, and catalytic properties of aluminas. In: Jentoft FC, editor. Advances in catalysis. Vol. 57. San Diego (CA): Academic Press; 2014. p. 319–404. doi: 10.1016/B978-0-12-800127-1.00003-5

[174] Trueba M, Trasatti SP. γ-alumina as a support for catalysts: a review of fundamental aspects. Eur J Inorg Chem. 2005;2005(17):3393–3403. doi: 10.1002/ejic.200500348

[175] Digne M, Sautet P, Raybaud P, et al. Use of DFT to achieve a rational understanding of acid–basic properties of γ-alumina surfaces. J Catal. 2004;226(1):54–68. doi: 10.1016/j.jcat.2004.04.020

[176] Cai SH, Rashkeev SN, Pantelides ST, et al. Atomic scale mechanism of the transformation of γ-alumina to θ-alumina. Phys Rev Lett. 2002;89(23):235501. doi: 10.1103/PhysRevLett.89.235501 12485016

[177] Greiner E, Kumar K, Sumit M, et al. Adsorption of l-glutamic acid and l-aspartic acid to γ-Al_2_O_3_. Geochim Cosmochim Acta. 2014;133:142–155. doi: 10.1016/j.gca.2014.01.004

[178] Zheng TT, Sun ZX, Yang XF, et al. Sorption of phosphate onto mesoporous γ-alumina studied with in-situ ATR-FTIR spectroscopy. Chem Cent J. 2012;6(1):26. doi: 10.1186/1752-153X-6-26 22472205 PMC3441211

[179] Mei D. First-principles characterization of formate and carboxyl adsorption on stoichiometric CeO_2_(111) and CeO_2_(110) surfaces. J Energy Chem. 2013;22(3):524–532. doi: 10.1016/S2095-4956(13)60069-8

[180] Wu B, Imc L. Surface functional group engineering of CeO_2_ particles for enhanced phosphate adsorption. Environ Sci Technol. 2020;54(7):4601–4608. doi: 10.1021/acs.est.9b06812 32182042

[181] Bhasker-Ranganath S, Zhao C, Xu Y. Theoretical analysis of the adsorption of phosphoric acid and model phosphate monoesters on CeO_2_(111). Surf Sci. 2021;705:121776. doi: 10.1016/j.susc.2020.121776

[182] Wang ZG, Bi WZ, Ma SC, et al. Facet-dependent Effect of well-defined CeO_2_ nanocrystals on the adsorption and dephosphorylation of phosphorylated molecules. Particle Particle Syst Charact. 2015;32(6):652–660. doi: 10.1002/ppsc.201400230

[183] Patil S, Sandberg A, Heckert E, et al. Protein adsorption and cellular uptake of cerium oxide nanoparticles as a function of zeta potential. Biomaterials. 2007;28(31):4600–4607. doi: 10.1016/j.biomaterials.2007.07.029 17675227 PMC2259388

[184] De Faria LA, Trasatti S. The point of zero charge of CeO_2_. J Colloid Interface Sci. 1994;167(2):352–357. doi: 10.1006/jcis.1994.1370

[185] Li W, Deng Q, Fang G, et al. Facile synthesis of Fe_3_O_4_@TiO_2_–ZrO_2_ and its application in phosphopeptide enrichment. J Mater Chem B. 2013;1(14):1947–1961. doi: 10.1039/C3TB20127A 32260908

[186] Bai Q, Liu Y, Wang Y, et al. Protein separation using a novel silica-based RPLC/IEC mixed-mode stationary phase modified with N-methylimidazolium ionic liquid. Talanta. 2018;185:89–97. doi: 10.1016/j.talanta.2018.03.042 29759254

[187] Guo X, Bai H, Chen L. Imidazole-octyl mixed-mode stationary phase based on macroporous silica for the purification of ovomucoid and ovotransferrin. Mikrochim Acta. 2023;190(10):404. doi: 10.1007/s00604-023-05986-7 37728672

[188] Nagase K. Innovative bioseparation technologies employing thermoresponsive polymer interfaces. Polym J. 2026;58(3):181–205. doi: 10.1038/s41428-025-01117-6

